# Tailoring Ion Transport in Li_3‐3y_Ho_1+y_Cl_6‐x_Br*_x_* via Transition‐Metal Free Structural Planes and Charge Carrier Distribution

**DOI:** 10.1002/advs.202409668

**Published:** 2024-12-17

**Authors:** Bright O. Ogbolu, Tej P. Poudel, Thilina N. D. D. Dikella, Erica Truong, Yudan Chen, Dewen Hou, Tianyi Li, Yuzi Liu, Eric Gabriel, Hui Xiong, Chen Huang, Yan‐Yan Hu

**Affiliations:** ^1^ Department of Chemistry and Biochemistry Florida State University Tallahassee FL 32306 USA; ^2^ Materials Science and Engineering Program Florida State University Tallahassee FL 32310 USA; ^3^ Micron School of Materials Science and Engineering Boise State University Boise ID 83725 USA; ^4^ Center for Nanoscale Materials Argonne National Laboratory Argonne IL 60439 USA; ^5^ X‐Ray Science Division Argonne National Laboratory Argonne IL 60439 USA; ^6^ Department of Scientific Computing Florida State University Tallahassee FL 32306 USA; ^7^ Center of Interdisciplinary Magnetic Resonance National High Magnetic Field Laboratory Tallahassee FL 32310 USA

**Keywords:** all‐solid‐state batteries, halide solid electrolytes, high‐resolution XRD analysis, lithium deficiency, mixed‐anion, nuclear magnetic resonance, superionic conductor

## Abstract

Localized atomistic disorder in halide‐based solid electrolytes (SEs) can be leveraged to boost Li^+^ mobility. In this study, Li^+^ transport in structurally modified Li_3_HoCl_6_, via Br^−^ introduction and Li^+^ deficiency, is explored. The optimized Li_3‐3_ *_y_* Ho_1+_ *_y_* Cl_6‐_ *_x_* Br*_x_* achieves an ionic conductivity of 3.8 mS cm^−1^ at 25 °C, the highest reported for holmium halide materials. ^6,7^Li nuclear magnetic resonance and relaxometry investigations unveil enhanced ion dynamics with bromination, attaining a Li^+^ motional rate neighboring 116 MHz. X‐ray diffraction analyses reveal mixed‐anion‐induced phase transitions with disproportionate octahedral expansions and distortions, creating Ho‐free planes with favorable energetics for Li^+^ migration. Bond valence site energy analysis highlights preferred Li^+^ transport pathways, particularly in structural planes devoid of Ho^3+^ blocking effects. Molecular dynamics simulations corroborate enhanced Li^+^ diffusion with Br^−^ introduction into Li_3_HoCl_6_. Li‐Ho electrostatic repulsions in the (001) plane presumably drive Li^+^ diffusion into the Ho‐free (002) layer, enabling rapid intraplanar Li^+^ motion and exchange between the 2d and 4h sites. Li_3‐3_ *_y_* Ho_1+_ *_y_* Cl_6‐_ *_x_* Br*_x_* also demonstrates good battery cycling stability. These findings offer valuable insights into the intricate correlations between structure and ion transport and will help guide the design of high‐performance fast ion conductors for all‐solid‐state batteries.

## Graphical abstract

Mixed‐anion‐induced phase transitions, vacancy formation, and polyhedral distortion in Li_3‐3y_Ho_1+y_Cl_6‐ x_Br_x_ (0 ≤ x ≤ 3; 0 ≤ y ≤ 0.09) promote efficient Li^+^ ion transport. These materials exhibit high‐rate performance and good cycling stability when utilized in solid‐state batteries, offering promising prospects for advanced energy storage applications.

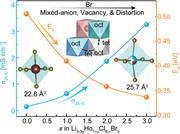

## 1 Introduction

There is a growing demand for energy‐dense storage systems, partly driven by the automotive industry's quest to make safe, energy‐efficient, long‐range electric vehicles.^[^ ^1^, ^2^ ^]^ As conventional lithium‐ion batteries, utilizing liquid electrolytes, are approaching their theoretical limits in energy and power density,^[^ ^3^, ^4^ ^]^ all‐solid‐state batteries (ASSBs), which employ solid electrolytes (SEs), are considered promising alternatives. The switch to SEs offers advantages, such as increased energy density facilitated by enabling high‐energy electrodes, reduced cost, and improved safety and thermal stability.^[^ ^5^, ^6^, ^7^, ^8^, ^9^, ^10^ ^]^

However, several significant challenges impede the successful deployment of ASSBs. One such challenge involves establishing efficient ion diffusion pathways within cathodes. Many cathode materials exhibit low ionic conductivities,^[^ ^11^, ^12^, ^13^ ^]^ thereby limiting power densities. This limitation is addressed in the current‐generation rechargeable batteries by the inter‐diffusion of liquid electrolytes into the cathode matrix. A similar strategy for ASSBs involves blending the active cathode materials with a fast ion conductor that possesses electrochemical stability at elevated voltages and chemical stability when in contact with cathode materials of interest. Among the various inorganic fast ion conductors, halide‐based ones have shown promising attributes, including robust oxidation stability, compatibility with high‐voltage cathode materials, wide band gap, deformability, and good ionic conductivity with a few surpassing 1 mS cm^−1^.^[^ ^14^, ^15^, ^16^, ^17^ ^]^ However, further improvement in the ionic conductivity is necessary for enhanced power density of ASSBs.

Asano et al. first reported a high lithium‐ion conductivity, 1.7 mS cm^−1^, for the lithium ternary halide Li_3_YBr_6_.^[^ ^18^ ^]^ Since then, expansive research has been conducted to understand the structural intricacies and ion transport mechanisms of these rare‐earth halide SEs.^[^ ^19^, ^20^ ^]^ Despite their earth‐abundance concerns, rare earth elements find extensive application in the industry due to their distinctive electronic configurations, variable valence states, and diverse utility options.^[^ ^21^, ^22^ ^]^ More importantly, Li_3_MX_6_ compounds are currently being explored as separators, catholyte components, grain boundary modifiers for oxide SEs,^[^ ^23^ ^]^ and protective coatings for superionic thiophosphate SEs against cathode‐active materials.^[^ ^24^ ^]^

Using density functional theory (DFT) and Ab Initio Molecular dynamics (AIMD) calculations, Wang et al. predicted isomorphs with good phase stability and high ionic conductivity, wherein Y^3+^ is substituted with other M^3+^ cations, including Ho^3+^ and Sc^3+^_._^[^ ^25^ ^]^ The calculated ionic conductivity for Li_3_HoCl_6_ (LHC) and Li_3_HoBr_6_ were 21 and 3.8 mS cm^−1^, respectively, at 300 K. They also suggested that a mixed anion framework in Li_3_MX_6_ produces disorder, which potentially benefits Li‐ion conduction. Furthermore, halide substitution provides a means to modify the polarizability and mechanical properties of Li_3_MX_6_.^[^ ^26^, ^27^, ^28^ ^]^ Solid electrolytes characterized by softer and more polarizable frameworks typically possess lower activation barriers and higher ionic conductivities. This correlation arises from the effective screening of mobile ion charges by more polarizable anion species, leading to weakened interactions between the mobile ions and the host framework.^[^ ^29^, ^30^ ^]^ Recent experimental efforts on Li_3_HoX_6_ (X = Cl, Br, or I) typically reported ≈1 mS cm^−1^ ionic conductivities,^[^ ^21^, ^31^, ^32^ ^]^ except for monoclinic, C2/m, Li_3_HoBr_3_I_3_ with an ionic conductivity of ≈2.7 mS cm^−1^.^[^ ^33^ ^]^ These experimental results have not reached the theoretical predictions. Moreover, the structural attributes that lead to optimal ionic conductivity in Li_3_HoX_6_ remain largely unknown. While the highest ionic conductivities are often reported for the monoclinic Li_3_HoBr_6_ phase with a cubic close‐packed (ccp) anion framework, optimal Li‐ion conductivities of 1.3 mS cm^−1^ have been demonstrated for orthorhombic Li_2.73_Ho_1.09_Cl_6_^[^ ^32^ ^]^ and ≈1.2 mS cm^−1^ for a hexagonal Li_3_HoCl_4_Br_2_ phase.^[^ ^34^ ^]^ Hence, there is a need for more rigorous investigations of these lithium holmium halide configurations.

Given the encouraging computational forecasts concerning Li_3_HoX_6_ (X = Cl, Br, or I), coupled with observed disparities in material performance and existing gaps in understanding structure‐ion transport relationships, our investigation delves into the collective impacts of anion mixing and Li deficiency on the Li_3_HoCl_6_ structure and its derivatives, Li_3‐3_ *_y_* Ho_1+_ *_y_* Cl_6‐_ *_x_* Br*_x_* (0 ≤ *x* ≤ 3; 0 ≤ *y* ≤ 0.09). We examined how diverse structural variables influence ion transport at both macroscopic and microscopic scales. We obtained a record room‐temperature ionic conductivity of ≈3.3 and ≈3.8 mS cm^−1^ for the stoichiometric Li_3_HoCl_3_Br_3_ (LHCB) and Li‐deficient Li_2.73_Ho_1.09_Cl_3.27_Br_2.73_ (def‐LHCB) monoclinic phases, respectively. This experimental outcome is consistent with the 3.8 mS cm^−1^ calculated for the monoclinic Li_3_HoBr_6_ isomorph and is over 20 times greater than the Li_3_HoCl_6_ trigonal phase (≈0.16 mS cm^−1^). Our multi‐modal characterization, using synchrotron x‐ray diffraction (SXRD), high‐resolution solid‐state nuclear magnetic resonance (NMR), ab initio molecular dynamics simulations (AIMD), bond valence site energy (BVSE) calculations, and electrochemical impedance spectroscopy (EIS), showed that anion mixing and adjustments in mobile ion concentration resulted in disproportionate polyhedral volume expansion, octahedral distortion, increased vacancy concentration, improved transport pathways, and reduced activation energy barriers. As a catholyte, both LHCB and def‐LHCB demonstrated excellent chemical interface stability with the TiS_2_ cathode active material. They achieved good cycling stability, retaining ≈95% and 90% of their capacities, respectively, after 70 cycles.

## 2 Results and Discussion

### 2.1 The Trigonal Structure of Li_3_HoCl_6_

**Figure 1:**
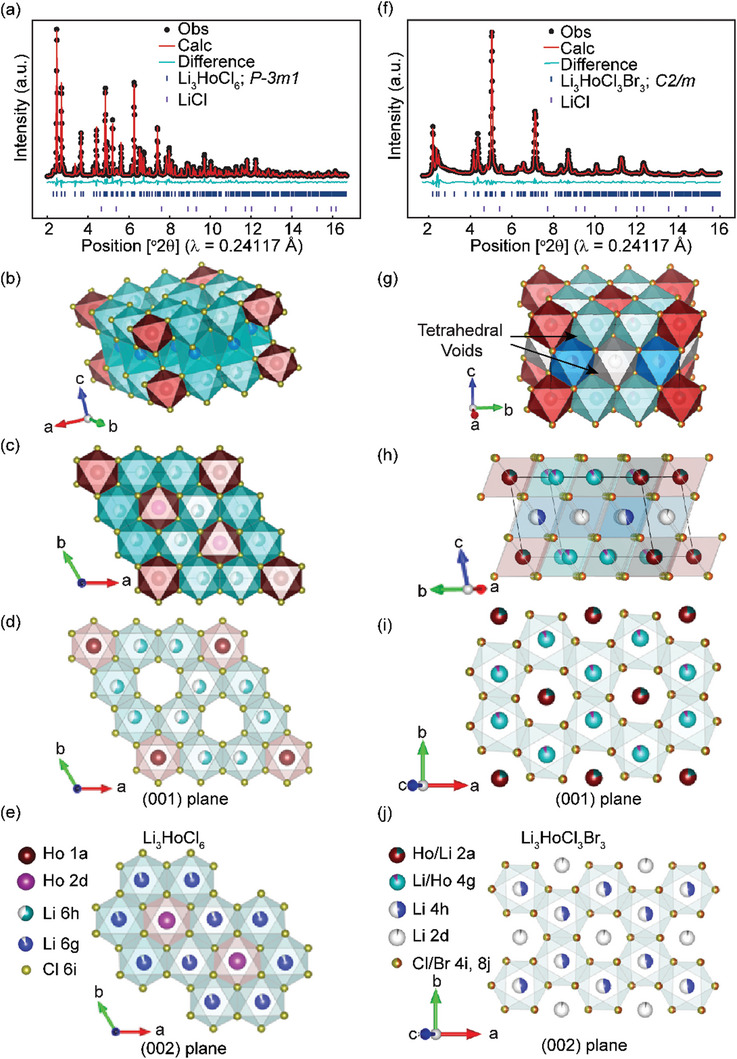
High‐resolution XRD data and the refined crystal structures of Li_3_HoCl_6_ and Li_3_HoCl_3_Br_3_. (a) High‐resolution XRD and refinement of Li_3_HoCl_6_. (b) Trigonal Li_3_HoCl_6_ structure. (c) The arrangement of edge‐sharing (LiCl_6_)^5−^ octahedra in Li_3_HoCl_6_ forms a honeycomb‐like network viewed from the c‐direction. The (001)‐plane caps the top layer. (d) Isolated (001)‐plane in Li_3_HoCl_6_, revealing octahedra voids. Octahedra are made more transparent to visualize the cations better. (e) Cut out of the (002)‐plane in Li_3_HoCl_6_. (HoCl_6_)^3−^ octahedra are encircled in the honeycomb motif of the (LiCl_6_)^5−^ octahedra. (f) Refinement of the high‐resolution XRD for Li_3_HoCl_3_Br_3_ and (g) the refined monoclinic Li_3_HoCl_3_Br_3_ structure (h) Stacking of the (001) and (002) lattice planes in Li_3_HoCl_3_Br_3_, (i) cation ordering in the (001)‐plane, and (j) (002)‐plane in Li_3_HoCl_3_Br_3_.

The trigonal (P3̅m1) Li_3_HoCl_6_ unit cell consists of a total of four octahedrally coordinated cation sites, namely Li1 at Wyckoff 6 g and Ho1 at Wyckoff 1a in the (001)‐plane, and Li2 at Wyckoff 6h and Ho2 at Wyckoff 2d in the (002)‐plane. The (001, 002) planes exhibit a honeycomb‐like pattern formed by the arrangement of six LiCl_6_^5−^ edge‐sharing octahedra (**Figure** **1**a–e). HoCl_6_^3−^ octahedra (Wyckoff 1a) occupy the corners of the unit cell within the (001) plane but are found missing in the center spots surrounded by six‐connected LiCl_6_^5−^, creating octahedra voids (Figure 1c). In the (002)‐plane, all the voids created by the six connected LiCl_6_^5−^ octahedra (Wyckoff 6h) are occupied by HoCl_6_^3−^ (Wyckoff 2d, Figure 1d). The (001, 002) planes are stacked along the *c*‐direction (Figure 1b–e). Lithium forms face‐sharing sequences along the *c*‐direction and edge‐sharing network within the *ab*‐plane, creating tetrahedral interstitial sites.

### 2.2 The Monoclinic Structure of Li_3‐3y_Ho_1+y_Cl_6‐x_Br_x_ (x ≥ 2, y = 0 or 0.09)

LHCB with higher bromine contents (*x* ≥ 2) adopts the monoclinic structure (Figure 1f–j). In the monoclinic structure (space group C2/m), holmium mainly occupies the 2a Wyckoff positions, and lithium occupies the Wyckoff 4g sites in the (001)‐lattice plane. Within the (001)‐plane, a distinctive mixing of Li/Ho octahedral is apparent, giving rise to cation site disorder within the layer (Figure 1i).^[^ ^33^, ^35^ ^]^ The (002)‐lattice plane is Ho‐free, consisting of partially occupied LiCl_6_^5−^ edge‐shared octahedra in the 4h Wyckoff positions forming hexagonal rings around the 2d octahedra sites (Figure 1h,j). All octahedra within each stacked lattice plane are interconnected at their edges in all directions. This edge‐sharing connectivity between octahedra creates tetrahedral interstitial sites through which Li^+^ ions must pass to reach the stable octahedral positions. The lithium sites, characterized by a multiplicity of four (4g, 4h), create a honeycomb‐like network around isolated octahedral sites with lower multiplicities (2a, 2d).^[^ ^35^ ^]^ When analyzing the planar orientation LHCB across several unit cells along the *c*‐direction, an almost random stacking arrangement becomes evident,^[^ ^33^, ^36^ ^]^ as illustrated in Figure S1 (Supporting Information).

The def‐LHCB, lithium removal generates more Li^+^ vacancies, which is beneficial for enhanced ion mobility. Extra holmium is introduced for charge balance, which we posit to increase the intralayer entropy and electrostatic repulsions in the Li/Ho‐shared (001)‐plane, driving Li^+^ into adjacent Ho‐free (002)‐layers and enhancing Li^+^ transport.

### 2.3 Phase Transitions from Trigonal to Monoclinic in Li_3‐3y_Ho_1+y_Cl_6‐x_Br_x_ (x ≥ 2, y = 0 or 0.09)

The laboratory X‐ray diffraction patterns for the stoichiometric Li_3_HoCl_6‐_ *_x_* Br*_x_* series show a phase transition from the trigonal Li_3_HoCl_6_ phase, space group: P3̅m1, to the monoclinic phase (space group: C2/m) with increased Br substitutions (*x* ≥ 2), as shown in Figure S2 (Supporting Information). The major reflections shift slightly to lower 2θ values with Br → Cl substitution (Figure S2a, Supporting Information) in the P3̅m1 and C2/m phases, implying an expansion of the crystal lattice by incorporating the larger‐radius Br^−^. Lab x‐ray diffraction (XRD) patterns for Li deficient samples Li_2.73_Ho_1.09_Cl_6‐_ *_x_* Br*_x_*, shown in Figure S2b (Supporting Information), are generally not different from the stoichiometric compositions, except for the first member (*x* = 0) of the deficient analog, Li_2.73_Ho_1.09_Cl_6,_ which showed reflections that could be assigned to an orthorhombic crystal system (space group: Pnma).^[^ ^32^ ^]^ The trigonal phase was re‐established upon the introduction of Br (*x* = 1), and with further addition of Br (*x* ≥ 2), only the monoclinic‐like reflections were observed.^[^ ^37^, ^38^ ^]^ The synthesized compounds all have a high phase purity, with LiCl as a minor impurity accounting for ≈1%.

**Figure 2:**
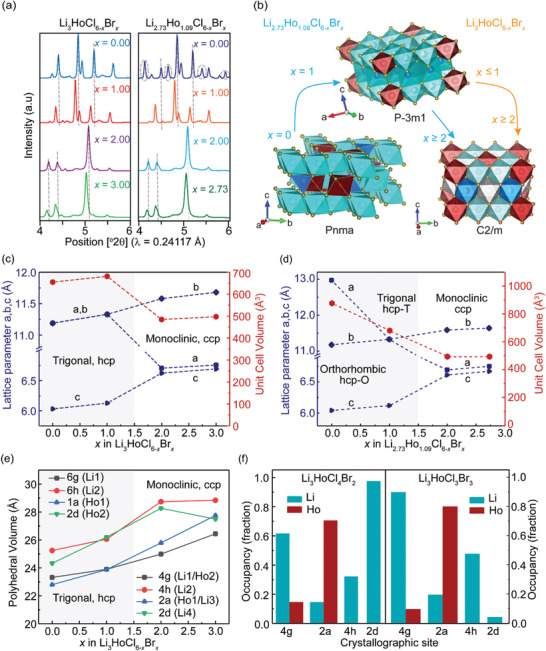
Bromination‐induced structural transitions of the Li_3_HoCl_6‐_ *_x_* Br*_x_* (0 ≤ *x ≤* 3) and Li_2.73_Ho_1.09_Cl_6‐_ *_x_* Br*_x_* (0 ≤ *x ≤* 2.73) series. (a) Magnified view of select high‐resolution X‐ray 2θ ranges. Peak shifts, attenuations, and appearances confirm phase changes. (b) Illustration of the structure transformations upon bromine substitution. (c,d) The structure transitions from the hcp to the ccp anion framework with increased bromination. The lattice parameter *a* decreases while *b* and *c* increase. (e) Polyhedral volume as a function of bromine substitution shows a nonlinear increase in the octahedra sizes and (f) Cation site ordering for Li_3_HoCl_4_Br_2_ and Li_3_HoCl_3_Br_3_. A marked variation in Li^+^ occupancies at 2d hints at a highly mobile site. Similar changes were observed in the Li‐deficient series, as shown in Figure S6 (Supporting Information).

Synchrotron XRD data confirms bromination‐induced phase transformation in Li_3_HoCl_6‐_ *_x_* Br*_x_*, characterized by peak shifts, peak intensity attenuation, and new peak appearances, as shown in **Figure** **2**a. An illustration of the structural changes for the series is presented in Figure 2b. Upon careful examination of the high‐resolution PXRD pattern for these monoclinic structures, triangular Warren‐type peak shapes are observed between the 2 and 3° 2θ range (Figure S3, Supporting Information), indicating the presence of planar defects.^[^ ^39^, ^40^, ^41^ ^]^ The reduction in reflection intensities observed for the monoclinic systems (*x ≥* 2) compared to the Li_3_HoCl_6_ trigonal reflections can be attributed to the structural transition from a higher to a lower symmetry crystal system, characterized by a much smaller unit cell dimension. Rietveld refinements against the high‐resolution diffraction data (Figure 1; Figure S4 and S5, Supporting Information) were used to study detailed structural changes upon bromine incorporation.

### 2.4 Changes in Lattice Parameters

The lattice parameter is a key descriptor that captures structural transformations. The transition from trigonal to monoclinic phase is simultaneously accompanied by transforming the anion sublattice from hexagonal close packing (hcp) to cubic close packing (ccp).^[^ ^35^, ^42^ ^]^ For the hcp frameworks (*x* ≤ 1), the increase in unit cell volume corresponds to the increase in *a*‐ and *c*‐ lattice parameters (Figure 2c). Further increase in the Br content (*x* ≥ 2) resulted in a reduction in unit cell volume. The reduction in volume appears to be primarily influenced by the compression of the unit cell along the *a*‐direction, while the lattice parameters *b* and *c* increase in comparison to the hcp structure. Nonetheless, within the monoclinic (ccp) phase (*x* ≥ 2), a general increase in the length of *a*, *b*, and *c* was observed (Figure 2c) with increased Br content in Li_3_HoCl_6‐_ *_x_* Br*_x_*. These observed linear expansions of the lattice within the same crystal system correlate with the degree of Br substitution, adhering to Vegard's law.^[^ ^43^ ^]^ Similar trends were observed for the Li‐deficient series (Figure 2d).

### 2.5 Polyhedral Distortion

The local coordination environments of Li/Ho show a contrasting trend to the size contraction observed for the hcp to ccp transition. A gradual non‐linear increase in polyhedral volumes is observed as the Br amount increases (Figure 2e). This is expected, given the ionic radius of bromide (1.96 Å) is greater than chloride (1.81 Å).^[^ ^44^ ^]^ For each composition, however, the polyhedral sizes are different. On average, the lithium octahedral within the (002) layer (Wyckoff 6h, 2d, and 4h positions) are larger in volume in comparison to the (001)‐layer octahedral (Wyckoff 6g, 4g, and 2a positions). This trend is observed (Figure 2e), particularly for the monoclinic structure, indicating an unequal distortion of the crystallographic sites in the different layers,^[^ ^45^ ^]^ which can benefit ion conduction.^[^ ^46^ ^]^ The holmium polyhedra in the (001)‐layer (Wyckoff 1a, 2a, and 4g positions) have the smallest polyhedron size, likely due to the stronger Coulombic attraction between Ho^3+^ and the surrounding halide ions.^[^ ^31^, ^33^ ^]^ Excess holmium in def‐LHCB creates a more distorted polyhedral environment.

To better understand the local structure and bonding arrangements, the pair distribution function (PDF) was calculated for Li_3_HoCl_6‐x_Br*_x_* (0 ≤ *x ≤* 3) using the refined crystal structure determined from the high‐resolution XRD data. PDF provides insights into the spatial arrangement of atoms by quantifying the probability of finding pairs of atoms at different distances.^[^ ^47^ ^]^ As shown in the PDF *G(r)* overlay (Figure S7, Supporting Information), the atom pairs ≈2.6 Å (Li/Ho‐X) and 3.7 Å (X‐X; where X = Cl/Br), which constitute the local coordination environments, readily extend to higher *r* values (atom‐atom distance) with increased Br^−^ incorporation. This longer Li/Ho‐X distance corroborates the observed increase in polyhedral volume derived from the refined structure analysis.

### 2.6 Intralayer Li/Ho Site Ordering

Beyond the size variation among different polyhedra, cation site ordering plays a crucial role in influencing the diffusion behavior of lithium ions.^[^ ^26^ ^]^ As the bromine content increases, intralayer cation site disorder emerges, characterized by mixed Li/Ho occupancies within the (001)‐layer (Figure 2f). It is noteworthy that adding Ho to the Li 4g site and Li to the 2a site improved the model's 2–3° 2θ reflection intensities and the overall refinement quality significantly for x ≥ 2 compositions (Figures S4 and S5, Supporting Information), a strong indication of the presence of intralayer cation site mixing. A key difference between the trigonal and monoclinic LHCB structures is that the monoclinic structure has shared Li/Ho occupancies at the Wyckoff 4a and 2a sites. In contrast, all Li and Ho sites are distinct in the trigonal structure (Figure 1d,e). An increase in the degree of intralayer cation disorder from 0% for Li_3_HoCl_6_ to ≈20% [1:4 Li/Ho occupancy] at the 2a site and 10% [1:9 Li/Ho occupancy] at the 4g sites in LHCB was observed (Figure 2f). For the def‐LHCB, occupancies of Li in the (001) plane (Wyckoff 4g) is ≈30%, with ≈20% Ho and ≈50% vacancy making up the remainder (Figure S6, Supporting Information). The (002)‐layer, including the 4h and 2d sites, is exclusively occupied by Li^+^, with significantly varied occupancies (Figure 2f; Figure S6, Supporting Information). Increased disorders, particularly those leading to a frustrated energy landscape, have been demonstrated to enhance ion transport.^[^ ^48^, ^49^, ^50^ ^]^

### 2.7 Particle Morphology and Planar Defects

**Figure 3:**
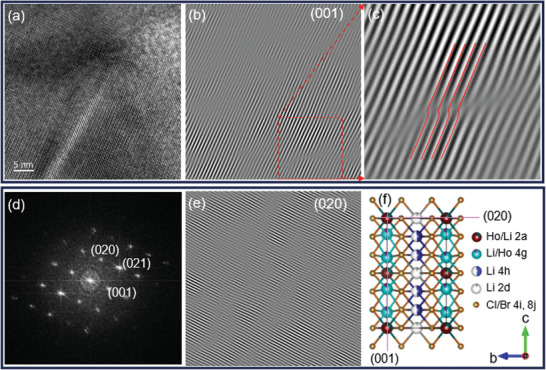
(a) High‐resolution TEM images of Li_3_HoCl_3_Br_3_. (b,e) inverse FFT of the (001) and (020) sequences revealing ample dislocations in the lattice fringes. (c) Magnified view of the dislocation defects highlighted by the red lines (d) The corresponding fast Fourier transform (FFT) pattern of image a. (f) The simulated model as seen down the [100] direction, the magenta lines emphasize the two planes of interest.

High‐resolution transmission electron microscopy (HRTEM) images for LHCB were acquired to examine the morphology, as shown in **Figure** **3**a. The corresponding fast Fourier transform (FFT) pattern with a detailed index plane displays clear diffraction spots (Figure 3d). The structure defects are visualized by the inverse fast Fourier transform (IFFT) (Figure 3b,e), with a focus on the (001) and (020) planes, and dislocation‐like structures become apparent upon magnification (Figure 3c). These large number of dislocations could be associated with stacking faults, which are also spotted in the XRD diffraction data. The planes of interest are highlighted in the simulated structure for better perspective (Figure 3e).

### 2.8 Li^+^ Local Structures and Dynamics Probed by High‐Resolution ^6,7^Li NMR

**Figure 4:**
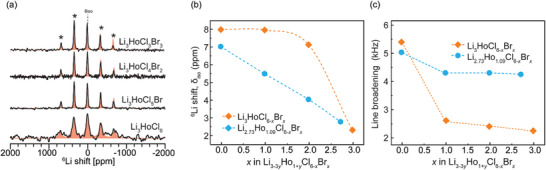
Local structures of Li_3_HoCl_6‐_ *_x_* Br*_x_* probed with high‐resolution ^6^Li NMR. (a) ^6^Li NMR spectra and (b) isotropic shift δ_iso_ as a function of *x* in Li_3_HoCl_6‐_ *_x_* Br*_x_* and Li_2.73_Ho_1.09_Cl_6‐_ *_x_* Br*_x_*. The isotropic chemical shift moves toward lower ppm, indicating changes in the lithium environment due to bromine addition. (c) The line broadening of the isotropic peak as a function of *x* in Li_3_HoCl_6‐_ *_x_* Br*_x_* and Li_2.73_Ho_1.09_Cl_6‐_ *_x_* Br*_x_*. The decrease in linewidth with increased bromine substitution suggests faster Li^+^ motion. The relatively broader linewidth for the Li‐deficient (blue) series indicates higher local structural disorder.

NMR shifts are highly sensitive to the surrounding nucleus environment. Changes in the NMR shifts are primarily ascribed to deviations in local magnetic fields induced by variations in chemical shielding. Notable shifts occur due to elemental substitutions, structural disorder, and dynamics.^[^ ^51^ ^]^ The ^6^Li magic‐angle‐spinning (MAS) NMR of the LHCB and def‐LHCB series exhibit manifolds of spinning sidebands (SSBs) due to paramagnetic interactions between Li and unpaired electrons in Ho^3+^ (**Figure** **4**a; Figure S8, Supporting Information). The shift of the isotropic peak for both the LHCB and def‐LHCB series moves toward lower ppm with increasing Br content (Figure 4b), suggesting changes in the local coordination environment that support successful bromine incorporation. Isotropic‐peak focused spectra are presented in Figure S8 (Supporting Information). The ^6^Li NMR shift changes with increasing Br content in the def‐LHCB series is gradual, while the major change occurs between *x* = 2 and 3 in the stoichiometric Li_3_HoCl_6‐_ *_x_* Br*_x_*. This contrast of ^6^Li shift change between Li_2.73_Ho_1.09_Cl_6‐_ *_x_* Br*_x_* and Li_3_HoCl_6‐_ *_x_* Br*_x_* echoes Li redistribution in the structures. For the stoichiometric Li_3_HoCl_6‐_ *_x_* Br*_x_*, significant Li shuffling at different crystallographic sites occurs (Figure 2f) between x = 2 and 3 compositions, leading to a relatively sharp change in the average ^6^Li NMR shift, while for the Li‐deficient Li_2.73_Ho_1.09_Cl_6‐_ *_x_* Br*_x_* series, the Li redistribution process is more subtle and gradually evolving (Figure S6, Supporting Information).

From the ^6^Li MAS NMR spectra (Figure 4a), individual Li sites are indistinguishable, perhaps due to fast Li site exchange during ion conduction. The shift anisotropy, an indicator of structural disorder, is calculated based on the intensities of the SSBs manifolds (Figure S8b). A larger shift anisotropy broadly connotes higher disorder, resulting from randomly oriented spins with varying local structural environments.^[^ ^51^, ^52^, ^53^ ^]^ Larger shift anisotropy is obtained for the Li deficient compositions Li_2.73_Ho_1.09_Cl_6‐_ *_x_* Br*_x_* in comparison to the stoichiometric Li_3_HoCl_6‐x_Br*_x_* series, implying increased local disorder at the Li‐sites of Li_2.73_Ho_1.09_Cl_6‐x_Br_x_. This finding of higher disorder in the def‐LHCB aligns with the structural analysis obtained from the X‐ray refinement results.

Figure 4c shows the linewidth evolution of the isotropic peak with bromine substitution. A general narrowing of the isotropic peak is observed for both series but is more conspicuous in the stoichiometric Li_3_HoCl_6‐x_Br*_x_* series. Fundamentally, the NMR peak broadening can be attributed to two distinct mechanisms: homogeneous and inhomogeneous broadening, each associated with specific characteristics of local field interactions. Homogeneous broadening is induced by the presence of randomly fluctuating local fields of spins, resulting in a range of energy levels and frequencies.^[^ ^51^, ^54^ ^]^ While inhomogeneous broadening originates from the distribution of the resonant frequency generated by dispersion of the magnetic field^[^ ^55^ ^]^ shifting the energies of the Zeeman eigenstates – the quantized energy levels associated with the magnetic interactions. Whereas inhomogeneous broadening can be removed by magic angle spinning (MAS), with samples spun at an angle of 54.7° relative to the magnetic field $\vec{B}$_0,_^[^ ^56^ ^]^ homogeneous broadening can only be partially removed by MAS. The marked line broadening observed in Li_3_HoCl_6_ reflects a persistent homogeneous broadening of the isotropic peak. The narrowing of the isotropic peak with bromine substitution is likely an attribute of fast ion motion, which reduces line broadening due to motional averaging. The trend in measured ionic conductivity (vide infra) echoes the peak narrowing for the respective series.

**Figure 5:**
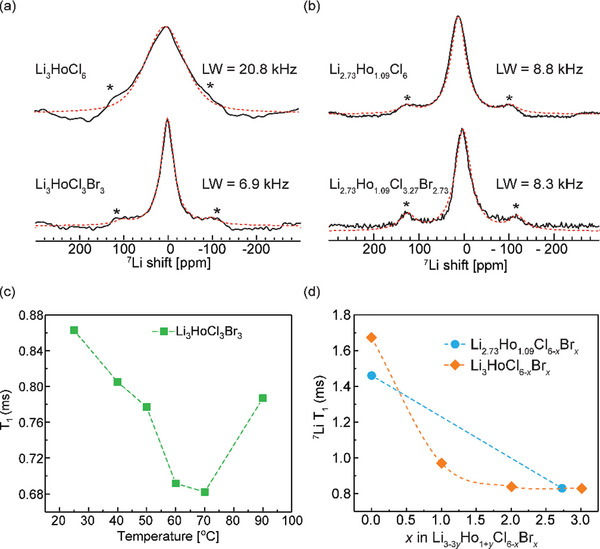
Li^+^ dynamics in Li_3‐3_ *_y_* Ho_1+_ *_y_* Cl_6‐_ *_x_* Br*_x_*, examined with ^7^Li NMR spectroscopy and relaxometry. (a) ^7^Li NMR spectra for Li_3_HoCl_6‐_ *_x_* Br*_x_* (b) ^7^Li NMR spectra for Li_2.73_HoCl_6‐_ *_x_* Br*_x._* The slight upfield shift in the ^7^Li spectra supports successful Br incorporation. The sharpening of the peaks in each series is associated with increased Li^+^ motion. (c) Variable‐temperature ^7^Li NMR T_1_ measurements on Li_3_HoCl_3_Br_3_. It indicates Li‐ion mobility is in the intermediate motion regime, with the minimum reached at ≈70 °C. (d) Spin‐lattice relaxation time (T_1_) evolution for both series at room temperature. T_1_ decreases with bromine substitution, suggesting enhanced Li^+^ conductivity. The asterisks in (a,b) denote the spinning side bands.

^7^Li MAS NMR results (**Figure** **5**a,b) agree with the insights gained from ^6^Li NMR. The shift of the ^7^Li resonances to lower ppm indicates successful bromine substitution. Likewise, spectral line narrowing within each series is observed with increasing Br content. The increase in peak sharpness, especially for the stoichiometric LHCB series, could indicate increased motion.^[^ ^57^ ^]^ On closer inspection, the ^7^Li resonance for def‐LHCB*_,_* 8.29 KHz, is significantly broader than its counter composition LHCB, 6.87 KHz. The observed broadening further supports the proposition of a notably higher disorder within the Li‐deficient series.^[^ ^58^ ^]^

### 2.9 Ion Dynamics Probed using NMR Relaxometry

To provide a more quantitative assessment of changes in Li^+^‐ion dynamics, we conducted measurements of the ^7^Li spin‐lattice (T_1_) relaxation time. Spin‐lattice relaxation time (T_1_) measures how quickly the nuclear spins return to their equilibrium state after external perturbation. The Bloembergen‐Purcell‐Pound (BPP) model,^[^ ^59^ ^]^ shown in Equation (1), correlates the spin‐lattice relaxation time T_1_ with ion mobility.

(1) $$\frac{1}{T_{1}}=\frac{3\mu_{o}^{2}\gamma^{4}\hbar^{2}}{10r_{o}^{6}}\;\;\left[\frac{\tau_{c}}{1+{\left(\omega_{o}\tau_{c}\right)}^{2}}+\frac{4\tau_{c}}{1+4{\left(\omega_{o}\tau_{c}\right)}^{2}}\right]$$

where τ_c,_ represents the mean residence time between two successive jumps of the Li^+^ ions, ω_o_ is the Larmor frequency, r_o_ is the interatomic distance, ℏ is the reduced Planck constant, γ is the magnetogyric ratio, and µ_o_ is the magnetic permeability of free space.

Li^+^ motion increases as T_1_ decreases in the slow‐motion region (ω_o_τ_c_ ≫ 1), while T_1_ increases with increased motional rate in the fast‐motion region (ω_o_τ_c_ ≪ 1). T_1_ minimum corresponds to the point where the motional rate is equivalent to the Larmor frequency (ω_o_τ_c_ ≈ 1). As shown in Figure 5c, variable‐temperature ^7^Li NMR T_1_ measurement was conducted on LHCB to establish the motional region. The trend displays decreasing T_1_ values with increased motional rate up until ≈70 °C where it reaches the T_1_ minimum, and a trend reversal ensues with further temperature increase. It can be inferred that the motion of Li^+^ ion in this material falls within the intermediate‐motion region of the BPP model, with a jump frequency of ≈116 MHz at 70 °C. Below 70 °C, smaller T_1_ values correspond to faster Li^+^ motion. Room‐temperature ^7^Li T_1_ relaxation measurement (Figure 5d) resulted in a shorter T_1_ of 0.83 ms for both LHCB and the def‐LHCB analog, compared to 1.67 ms for Li_3_HoCl_6_, indicating enhanced Li^+^ ion dynamics of the former. Interestingly, the T_1_ trend, especially for Li_3_HoCl_6‐_ *_x_* Br*_x_*, also mirrors the trend in the line broadening (Figure 4c). Sharper resonances, shorter T_1_, and higher ion mobility are correlated in this material class.

### 2.10 Enhanced Li^+^ Diffusion in Structures with Higher Br^−^ Content Revealed by AIMD Simulations

**Figure 6:**
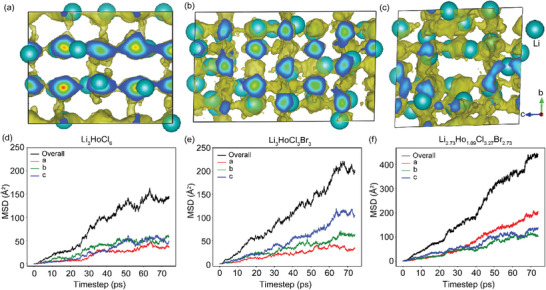
Li^+^ density distribution probability based on AIMD simulations at 900 K in (a) Li_3_HoCl_6_ in a 1 × 1 × 2 supercell, (b) Li_3_HoCl_3_Br_3_, 2 × 1 × 3 supercell, and (c) Li_2.73_Ho_1.09_Cl_3.27_Br_2.73,_ 2 × 1 × 2 supercell. All cells are displayed in the same orientation. Corresponding mean square displacements (MSD) of Li^+^ in (d) Li_3_HoCl_6_, (e) Li_3_HoCl_3_Br_3_, and (f) Li_2.73_Ho_1.09_Cl_3.27_Br_2.73._

To better understand the structure and Li^+^ ion transport relationship arising from Br^−^ incorporation in LHC, we conducted ab initio molecular dynamics (AIMD) simulations for LHC, LHCB, and def‐LHCB. The Li^+^ density probability maps, collected at 900 K, are shown in **Figure** **6**a–c. The AIMD simulation of Li^+^ trajectories (golden isosurfaces) in Li_3_HoCl_6_ shows a sparsely distributed diffusion pattern dominated by 1D interlayer diffusion along the *c* direction. Traces of possible diffusion in the other dimensions exist. However, this path along the *c*‐direction seems favored due to the face‐sharing octahedral network connecting the (001) and (002) layers, minimizing cation‐blocking effects from Ho^3+^ present in both planes. In contrast, a dense multidimensional distribution of Li^+^ diffusion paths is observed in LHCB (Figure 6b), resulting from structural modifications via Br^−^ incorporation, highlighted by expanded and distorted polyhedral geometries. Figure 6c shows an even denser spread of Li^+^ percolation paths in def‐LHCB, owing to more vacancies and occupational disorders at Li sites. This simulation data aligns with our experimental results, showing an ionic conductivity of def‐LHCB > LHCB ≫ LHC (vide infra).

The mean square displacements (MSD) of Li^+^, at 900 K, for LHC, LHCB, and def‐LHCB are shown in Figure 6d–f. The MSD plots for LHCB and def‐LHCB show significantly improved Li^+^ diffusion compared to LHC. The significant diffusion enhancement along the *c*‐direction (blue) in LHCB may arise from the electrostatic repulsions forcing Li^+^ out of shared Li/Ho sites in the (001) planes to the Ho^3+^ free (002) layer, where rapid diffusion along the *a* and *b* directions dominates. In the case of def‐LHCB, enhancement in diffusion along all three directions is observed, most prominently along the *a*‐direction, likely due to the additional repulsive effects caused by an excess of Ho in the (001) layer.

### 2.11 Transport Pathways Investigated using BVSE

**Figure 7:**
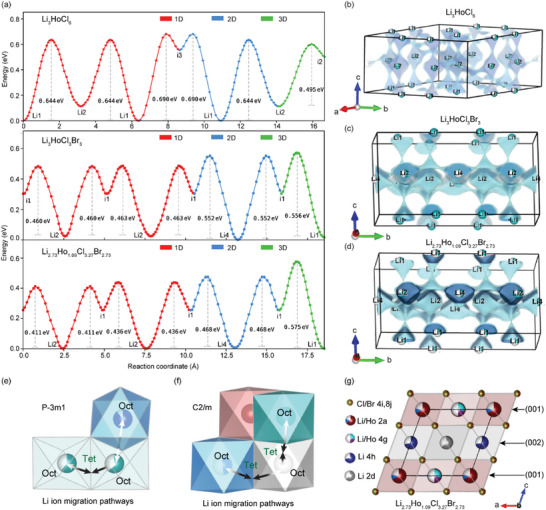
(a) Bond Valence Energy Landscape plots show the preferred migration pathways in Li_3_HoCl_6_, Li_3_HoCl_3_Br_3,_ and Li_2.73_Ho_1.09_Cl_3.3_Br_2.73._ The migration energy barrier is generally lower for the Br‐doped compositions than Li_3_HoCl_6_. (b‐d) Corresponding isosurfaces depicting potential Li^+^ trajectories in all three dimensions within the unit cells. The polyhedra are omitted for clarity. (e) the oct‐tet‐oct and oct‐oct Li^+^‐ion migration pathways in the trigonal structure. (f) the oct‐tet‐oct Li^+^‐ion migration pathway in the monoclinic frameworks. (g) the structure of Li_2.73_Ho_1.09_Cl_3.27_Br_2.73_, highlighting partial occupancies in both (001) and (002) planes.

In addition, we examined the lithium‐ion diffusion paths in the ordered LHC, LHCB, and def‐LHCB using bond valence energy landscape (BVEL) calculations to understand how local structural changes via anion mixing and Li^+^ removal impact ion conduction. From the bond valence energy landscape plots and isosurfaces shown in **Figure** **7**a–d, 3D ion diffusion is possible in all the compositions. However, the difference lies in the migration energy hurdles, which appear to be significantly lesser in the monoclinic (*x* ≥ 2) compositions. The observed phenomenon can be attributed to increased volume and vacancies (Figure 7g) facilitating ion percolation within frameworks with higher bromine content. Within the hcp system (*x* < 2), lithium hops can occur through face‐sharing oct‐oct, oct‐tet‐oct, and tet‐tet pathways.^[^ ^18^, ^25^ ^]^ Li^+^ diffusion is dominated by the direct oct‐oct jumps along the c‐direction, with a migration barrier of 0.644 eV in Li_3_HoCl_6_ (Figure 7a). Another possibility is the oct‐tet‐oct interstitial jumps within the *a‐b* plane (001) from an octahedral site to unoccupied neighboring tetrahedra interstitial sites^[^ ^28^ ^]^ and then to the next lower energy octahedra (0.690 eV). The tetrahedra sites, with relatively higher energy, serve as transition states for ionic conduction, as illustrated in Figure 7e,f. A potential Li2‐tet‐Li2 path within the (002) plane displays an energy barrier of 0.495 eV. However, the presence of holmium at the center of this plane is likely to induce cation‐blocking effects, hindering the free motion of Li^+^ ions.

In the ccp anion framework (x ≥ 2), only oct‐tet‐oct paths are present (Figure 7f).^[^ ^60^ ^]^ The oct‐tet‐oct path within the (002)‐plane exhibits the lowest energy for Li‐ion migration, 0.46 and 0.43 eV for the stoichiometric LHCB and highly disordered def‐LHCB, respectively (Figure 7a). This could arise from the absence of Ho^3+^ in the (002)‐planes, minimizing the cation‐blocking effect for this pathway. This dominant migration path is depicted in the isosurface structures, shown in Figure 7c,d. 3D Li‐ion diffusion within the ccp framework (x ≥ 2) is possible because lithium can exchange between the (001) and (002) lattice planes along the c‐direction through the partially occupied Ho2/ Li1 sites (see Figure 1) to the 4h or 2d Li sites. This interplanar diffusion network is likely forced by the Li‐Ho repulsions in the mixed sites, higher in Ho^3+^ excess def‐LHCB, contributing to the higher ionic conductivity observed for the monoclinic compositions.

### 2.12 Transport Properties and Battery Cell Performance

**Figure 8:**
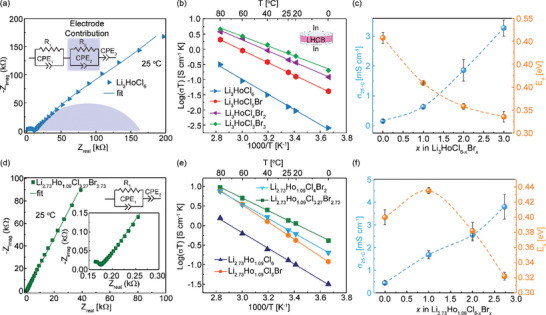
Ionic conduction properties in Li_3_HoCl_6‐_ *_x_* Br*_x_* and Li_2.73_Ho_1.09_Cl_6‐_ *_x_* Br*_x_*, determined with AC EIS. (a) and (d) the equivalent circuit fitting of the Nyquist plots of Li_3_HoCl_6_ and Li_2.73_Ho_1.09_Cl_3.27_Br_2.73_ (b,e) Arrhenius plots for Li_3_HoCl_6‐_ *_x_* Br*_x_* and Li_2.73_Ho_1.09_Cl_6‐_ *_x_* Br*_x_* series, respectively. (c,f) the conductivity at 25 °C and activation energy as a function of *x* in the Li_3_HoCl_6‐_ *_x_* Br*_x_* and Li_2.73_Ho_1.09_Cl_6‐_ *_x_* Br*_x_* series, respectively.

The ionic conductivity of LHCB is determined with electrochemical impedance spectroscopy (EIS) using a symmetric Li^+^ ion blocking setup, In|LHCB|In as shown in the inset of **Figure** **8**. The impedance spectra of LHCB are composed of a single suppressed semicircle at high frequency and a sloped line at low frequency. The data was analyzed with two (RQ) + Q equivalent circuit models to quantitatively understand these processes and their contributions to the impedance in LHCB (Figures S9 and S10, Supporting Information). A resistor R connected in parallel to a constant phase element (Q) is represented by the symbol (RQ). A constant phase element was employed instead of a capacitor to account for heterogeneous surfaces between the indium electrodes and the pellet.^[^ ^61^ ^]^ The bulk and grain boundary contributions could not be deconvoluted even at −20 °C, as shown in Figures S9 and S10 (Supporting Information). Hence, the conductivities described here indicate total conductivities.

Extracted room‐temperature (RT) ionic conductivities from the fitted Nyquist plots as a function of the Br content (*x*) are shown in Figure 8c,f). Li_3_HoCl_6_ has an ionic conductivity value of 0.16 mS cm^−1^at room temperature. The ionic conductivity increases with the Br content in both the stoichiometric and Li‐deficient LHCB series, with the highest observed RT ionic conductivity values of 3.27 and 3.80 mS cm^−1^ for LHCB and def‐LHCB, respectively. This represents an over 20‐fold improvement in Li^+^ transport compared to Li_3_HoCl_6_, likely due to the more favorable transport pathways discussed previously. The slightly higher ionic conductivity obtained for the def‐LHCB could result from additional Li^+^ vacancies. With further increase in the Br content beyond *x* = 3 and *x* = 2.73 for Li_3_HoCl_6‐_ *_x_* Br*_x_* and Li_2.73_Ho_1.09_Cl_6‐_ *_x_* Br*_x,_* respectively, ionic conductivity values decline sharply (Figure S11, Supporting Information). Factors such as exceeding optimum lattice polarizability (anion lattices becoming too soft),^[^ ^29^ ^]^ lengthening of the jump distance of mobile ions between isolated octahedral,^[^ ^62^ ^]^ octahedral sites becoming unstable and inaccessible by Li ions at much larger volume,^[^ ^25^ ^]^ potentially induce this sharp decline in Li‐ion conduction with elevated Br substitution.

Variable‐temperature impedance spectroscopy experiments were conducted (Figure S12, Supporting Information) to elucidate the transport properties further and to gain insights into thermodynamic factors influencing ionic transport. The Arrhenius plots (Figure 8b,e) display a nearly linear variation over the measured temperature range of 0 to 80 °C, suggesting the absence of phase transitions or degradations within this temperature range.^[^ ^9^ ^]^ The activation energies of LHC, LHCB_,_ and def‐LHCB, derived from Arrhenius plots, are 0.51, 0.34, and 0.32 eV, respectively (Figure 8c,f). The lower activation energy barrier in LHCB and def‐LHCB enhances ionic conductivity, as supported by the favorable migration pathways discussed in the BVSE analysis section. The equations for calculating the ionic conductivity and activation energy are provided in the Supporting Information (Equations S1 and S2). Furthermore, Li_3‐3_ *_y_* Ho_1+_ *_y_* Cl_6‐_ *_x_* Br*_x_* displays a low electronic conductivity, ≈7.7 × 10^−9^ S cm^−1^ (Figure S13, Supporting Information), which ensures a negligible contribution of electron transport to the measured total conductivity.

**Figure 9:**
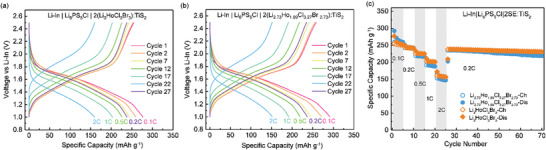
Cycling rate performance of Li_3_HoCl_3_Br_3_ and Li_2.73_Ho_1.09_Cl_3.27_Br_2.73_ solid electrolytes. Charge/discharge curves of (a) Li_3_HoCl_3_Br_3_ (b) Li_2.73_Ho_1.09_Cl_3.27_Br_2.73_, and (c) rate capability at 0.1, 0.2, 0.5. 1, and 2 C. C is the theoretical capacity of TiS_2_, 239 mAh g^−1^.

The galvanostatic rate performance of the ASSB half‐cells comprising 2LHCB:TiS_2_ catholyte, Li_6_PS_5_Cl separator, and Li‐In anode are shown in **Figure** **9**. The LHCB SE is used to provide fast Li^+^ migration due to its high ionic conductivity and oxidative stability. From the voltage profiles presented in Figure 9a,b, the LHCB and def‐LHCB half‐cells display an initial high discharge capacity of 259.5 and 271.4 mAh g^−1^ (Cycle 2) at 0.1 C, respectively.

This capacity is noticeably greater than the theoretical capacity of the cathode active material TiS_2_ (239 mAhg^−1^) and is likely due to potential capacity‐generating redox of unknown phases from SE and TiS_2_ reactions, as indicated in similar TiS_2_:SE catholyte studies.^[^ ^63^, ^64^, ^65^ ^]^ The rate performance of these cells was tested at 0.1, 0.2, 0.5, 1, and 2 C for five cycles each, followed by 45 cycles at 0.2 C (Figure 9c). The initial discharge capacities at 0.1, 0.2, 0.5, 1, and 2 C are 259.5, 244.0, 226.4, 203.3, and 159.9 mAh g^−1^, respectively, for LHCB. The corresponding values are 271.4, 244.9, 219.5, 191.8, and 152.0 mAh g^−1^, respectively, for def‐LHCB. Both cells demonstrated good retention capacity, with the LHCB cell performing slightly better than the def‐LHCB cell. After 70 cycles at 0.2 C, they maintained stable capacities of 230.7 and 219.5 mAh g^−1^, respectively, achieving capacity retentions of ≈95% for the LHCB cell and ≈90% for the def‐LHCB cell, indicating good cycling stability.

At a fast charge/discharge rate of 2C, LHCB and def‐LHCB half‐cells achieved high capacities of 159.9 and 152 mAh g^−1^ by the 27th cycle, respectively. This underscores their effectiveness in enabling rapid ion transport paths. The impressive capacity demonstrated by these half‐cells may also stem from improved utilization of TiS_2_ in the catholyte due to enhanced ion transport kinetics.

## 3 Conclusion

This work has synthesized Li_3‐3y_Ho_1+y_Cl_6‐x_Br*_x_* (0 ≤ *x* ≤ 3, 0 ≤ *y* ≤ 0.09), deciphered the structural origin of fast ion transport, and evaluated their performance as electrolytes in all‐solid‐state batteries. We focused on Li_3_HoCl_6_, stoichiometric Li_3_HoCl_3_Br_3,_ and Li deficient/Ho excess Li_2.73_Ho_1.09_Cl_3.27_Br_2.73,_ with RT ionic conductivities of 0.16, 3.3, and 3.8 mS cm^−1^, and E_a_ values of 0.51, 0.34, and 0.32 eV, respectively. Li_2.73_Ho_1.09_Cl_3.27_Br_2.73_ delivers the highest reported ionic conductivity for Ho‐based ternary halides. XRD refinement shows a structural transition from trigonal to monoclinic induced by Br^−^ incorporation in Li_3‐3y_Ho_1+y_Cl_6‐x_Br*_x_* and a significant redistribution of Li^+^ across varying compositions. Increasing Br^−^ in Li_3‐3y_Ho_1+y_Cl_6‐x_Br*_x_* expands the polyhedral sizes disproportionately, leading to octahedral distortion and wider bottlenecks that enhance Li^+^ diffusion. Removing Li^+^ tends to create a more disordered structure, and charge balancing with excess Ho^3+^ contributes to the distortion effects. ^7^Li NMR relaxometry, BVSE, and AIMD simulations corroborate enhanced Li^+^ dynamics and diffusion with Br^−^ introduction into Li_3_HoCl_6_. All‐solid‐state batteries employing Li_3‐3y_Ho_1+y_Cl_3_Br_3_ as electrolytes demonstrate good rate performance and cycling stability. This work provides new insights into how structural defects induced by tuning the anion framework influence the lithium sub‐lattice and ion transport in halide solid electrolytes. This contributes to the ongoing efforts in developing high‐performance solid electrolytes for all‐solid‐state batteries.

## 4 Experimental Section

### Synthesis

Lithium chloride (99.9% Sigma Aldrich) and lithium bromide (99.9% Sigma Aldrich) were dried at 200 °C for 12 h under a dynamic vacuum before use to remove any moisture. All the precursors were stored in an argon‐filled glovebox (VTI). A stoichiometric amount of LiCl, LiBr, and anhydrous HoCl3 (99.9% Alfa Aesar) was hand‐ground for 10 min using mortar and pestle to obtain a homogenous powder. The mixture was then quantitatively transferred into a quartz tube and sealed with an oxy‐hydrogen flame under a dynamic vacuum. The ampoules were heated to 650 °C at a ramping rate of ≈162.5 °C hr^−1^ in a box furnace (Thermo Scientific Lindberg/Blue M) and the temperature was held at 650 °C for 24 h. This was followed by a controlled slow cooling at ≈26 °C hr^−1^ to room temperature inside the box furnace. The co‐melted sample was then taken into the argon‐filled glovebox (MBroun), hand‐ground to powder for 15 min, and stored in glass vials for further characterization.

### Lab X‐ray Diffraction

Initial structure and phase composition characterization was performed using a Rigaku SmartLab X‐ray diffractometer in a Bragg–Brentano geometry with a Cu‐Kα radiation source (0.154 nm). The powder samples were packed in a zero‐background sample holder and sealed with Kapton film in an argon‐filled glovebox. Data was acquired in the range of 2θ values 10° to 80° at a step size of 0.03°_._

### Synchrotron X‐ray Diffraction

Synchrotron powder diffraction data was collected for the prepared Li_3‐3y_Ho_1+y_Cl_6‐_ *_x_* Br*_x_* samples at beamline 17‐BM‐B at the Advanced Photon Source, Argonne National Laboratory, with a measurement wavelength of 0.24117 Å. All the compositions were analyzed at room temperature. The instrument was calibrated using CeO_2_ as the standard. The 2D diffraction patterns were converted to 1D diffraction patterns by the GSAS‐ II software package.^[^ ^66^ ^]^

### Rietveld Refinement

The synchrotron X‐ray diffractograms were analyzed utilizing the GSAS‐II software package. The Li_3_YCl_6_ trigonal structure reported by Asano et al.^[^ ^18^ ^]^ was used to refine Li_3_HoCl_6_, and Y^3+^ was replaced with Ho^3+^_._ For the C2/m structures, *x* ≥ 2, insights were drawn from the Li_3_HoBr_6_ refinement reported by Plass et al.^[^ ^33^ ^]^ The general refinement steps outlined by Zeier and co‐workers^[^ ^45^, ^67^ ^]^ served as a guide. The background was refined using the chebyschev‐1 function. Then, the phase fractions, lattice, and instrument parameters were defined. The structure parameters–atomic coordinates, site occupancies, and displacement parameters (Uiso) of anions (Cl, Br) and the non‐mobile cation, Ho, were refined next. After several refinement cycles, the Cl/Br and Ho sites were constrained to their nominal stoichiometry, as their site occupancies remained unchanged. The same protocol was followed for the refinement of the mobile Li sites. All possible tetrahedral and octahedral vacancies and interstitial sites were tested for Li occupancies. The structural model with shared Li/Ho occupancies on the 4g and 2a sites within the (001)‐lattice plane proved the most stable and produced the lowest residual metrics. The synchrotron diffractogram fits and associated structural parameters were elaborated in the Supporting Information (Tables S1–S8 and Figures S4 and S5, Supporting Information). The polyhedral volume calculations and crystal structure visualization were done using the VESTA software.^[^ ^68^ ^]^ Stacking fault refinement was performed using the FAULTS program^[^ ^69^ ^]^ within the FullProf software suite.

### Pair Distribution Function Calculations

The PDF profile for the refined structure was calculated using the PDFgui software.^[^ ^70^ ^]^ The default calculation parameters (Scale factor, Qdamp, Qbroad) were retained, with the range adjusted to span from 1 to 6 Å to extract quantitative data regarding the length scale of local atomic arrangement.

### Transmission Electron Microscopy

TEM images were acquired using a JEOL JEM‐2100F microscope at a working voltage of 200 kV. To minimize the effect of air exposure, all samples were prepared in an argon‐filled glovebox.

### Solid‐State NMR

^6^Li and ^7^Li solid‐state NMR experiments were conducted using a Bruker Avance‐III spectrometer with an Ultrashield 500 MHz (11.74 T) 89 mm wide‐bore magnet. The Larmor frequencies of ^6^Li and ^7^Li were 73.6 MHz and 194.4 MHz, respectively. The sample powders were packed into 2.5 mm rotors made of ZrO_2_ under argon. The MAS rate was 25 kHz. Spin‐echo ^6^Li NMR experiments were performed with a π/2 pulse length of 6.9 µs and a recycle delay of 0.2 s. Projection magic‐angle turning and phase‐adjusted sideband separation (pjMATPASS)^[^ ^71^ ^] 7^Li NMR experiments were performed with a π/2 pulse length of 3.59 µs and a recycle delay of 0.15 s. Room temperature saturation recovery ^7^Li T_1_ measurements were conducted for Li_3_HoCl_6‐_ *_x_* Br*_x_* (*x* = 0, 1, 2 and 3) and Li_2.73_Ho_1.09_Cl_6‐_ *_x_* Br*_x_* (*x* = 0 and 2.73). ^7^Li NMR relaxometry measurements were conducted at elevated temperatures to study ion dynamics, utilizing a 300 MHz spectrometer tuned to the Larmor frequency of 116 MHz. Experiments were calibrated to solid LiCl at −1.1 ppm.

### Computational Approach

Density functional theory (DFT) calculations and ab initio Molecular Dynamics (AIMD) simulations were conducted using the Vienna Ab Initio Simulation Package (VASP) and the projector augmented wave (PAW) approach.^[^ ^72^, ^73^ ^]^ The Perdew–Burke–Ernzerhof generalized gradient approximation (GGA‐PBE) was used as the exchange‐correlation functional.^[^ ^74^ ^]^ The latest pseudopotential files provided by VASP were used. For configurations with site mixing and fractional occupancy, Python Materials Genomics (Pymatgen)^[^ ^75^ ^]^ was used to prescreen structures with different Li^+^/vacancy, Li^+^/Ho^+^, and Cl^−^/Br^−^ orderings based on the experimentally refined crystal structures. A handful of supercells were generated. Geometry optimizations were performed using DFT calculations with a kinetic energy cutoff of 520 eV. AIMD simulations were performed on the relaxed structures, using the canonical ensemble for 75 ps with a step time of 2 fs at a temperature of 900 K. A gamma‐point‐only sampling of k‐space and a plane‐wave energy cutoff of 280 eV were employed for all AIMD simulations.

### Bond Valence Sum Calculations

The bond valence site energy (BVSE) computations were obtained utilizing the softBV‐v131 algorithm.^[^ ^76^, ^77^ ^]^ Possible lithium diffusion pathways were estimated for the Li_3‐3y_Ho_1+y_Cl_6‐_ *_x_* Br*_x_* series with an auto‐calculated screening factor and a resolution of 0.1 Å. For compositions with the C2/m structure (*x* ≥ 2), it was impractical to execute the calculations in the presence of mixed cation occupancies. Hence, calculations were carried out following the removal of holmium from the 4g site and lithium from the 2a site. The holmium occupancy on the 2a site and lithium occupancies on the 4g site were adjusted to ensure charge neutrality. A similar approach had been reported elsewhere.^[^ ^35^ ^]^

### Electrochemical Measurements

The lithium‐ion conductivity of the Li_3‐3_ *_y_* Ho_1+_ *_y_* Cl_6‐_ *_x_* Br*_x_* series was assessed through AC impedance spectroscopy, employing a Biologic‐SP300 impedance analyzer. Cold‐pressed (500 psi) pellets with a thickness of ≈1.0 mm, a diameter of ≈6 mm, and a geometric density of ≈3.2 (±0.2) g cm^−3^ were used. Indium foils were applied onto the pellet's surface as blocking electrodes, and the pellet was situated in a bespoke cylindrical cell. Variable‐temperature impedance measurements were carried out using the CSZ Microclimate chamber, covering a temperature range from 0 to 80 °C. The measurements spanned frequencies from 5 MHz to 1 Hz, with an applied voltage of 10 mV. The DC polarization technique was applied to the samples in a symmetric setup In| Li_3‐3_ *_y_* Ho_1+_ *_y_* Cl_6‐_ *_x_* Br*_x_* |In to measure the electronic conductivity. Equilibrium currents were monitored at different voltages to ensure the accuracy of the electronic conductivity determination. Then, using Ohm's law (V = IR), the respective partial electronic conductivities were determined from the slope of the voltage vs current plot.^[^ ^78^ ^]^

### Assembly of ASSBs

Custom 10 mm split cells with stainless‐steel plungers as current collectors were utilized to fabricate all‐solid‐state battery half‐cells. To prepare the TiS_2_/ Li_3‐3_ *_y_* Ho_1+_ *_y_* Cl_6‐_ *_x_* Br*_x_* catholyte, TiS_2_ (Sigma, 99.9%) and the synthesized Li_3‐3_ *_y_* Ho_1+_ *_y_* Cl_6‐_ *_x_* Br*_x_* in a 1:2 (TiS_2_:SE) mass ratio was milled for 30 min to reduce particle size. Due to TiS_2_’s high electronic conductivity, no carbon additive was included. A Li_6_PS_5_Cl pellet prepared as described in previous reports^[^ ^57^ ^]^ was used as the separator. ≈12 mg of catholyte was then spread on one side of the pellet, yielding an areal loading of ≈1.25 mAh cm^−2^, and pressed at 300 MPa for 10 s. On the opposite side of the pellet, indium foil (≈0.313‐inch diameter) was positioned atop the pellet, followed by Li foil (≈0.188‐inch diameter) weighing roughly 1 mg. The cell was sealed tightly with vacuum grease and cycled under ≈30 MPa stack pressure at room temperature at the various C‐rates, operating within the 1 to 2.5 V voltage range versus Li–In.^[^ ^63^, ^79^ ^]^

## Conflict of Interest

The authors declare no conflict of interest.

## Supporting information



Supporting Information

## Data Availability

The data that support the findings of this study are available from the corresponding author upon reasonable request.
