# Supplementary material for: Tailoring Ion Transport in Li3‐3yHo1+yCl6‐xBr x via Transition‐Metal Free Structural Planes and Charge Carrier Distribution
Source: Adv Sci (Weinh). 2024 Dec 17;12(7):2409668. doi: 10.1002/advs.202409668 (PMC11831455; doi:10.1002/advs.202409668)
Supplement: Supplementary file 1 — Supporting Information [file ADVS-12-2409668-s001.docx]

Supporting Information

**Tailoring Ion Transport in Li_3-3y_Ho_1+y_Cl_6-x_Br*_x_* via Transition-Metal Free Structural Planes and Charge Carrier Distribution**

*Bright O. Ogbolu,^#^ Tej P. Poudel,^#^ Thilina N. D. D. Dikella, Erica Truong, Yudan Chen, Dewen Hou, Tianyi Li, Yuzi Liu, Eric Gabriel, Hui Xiong, Chen Huang, Yan-Yan Hu**


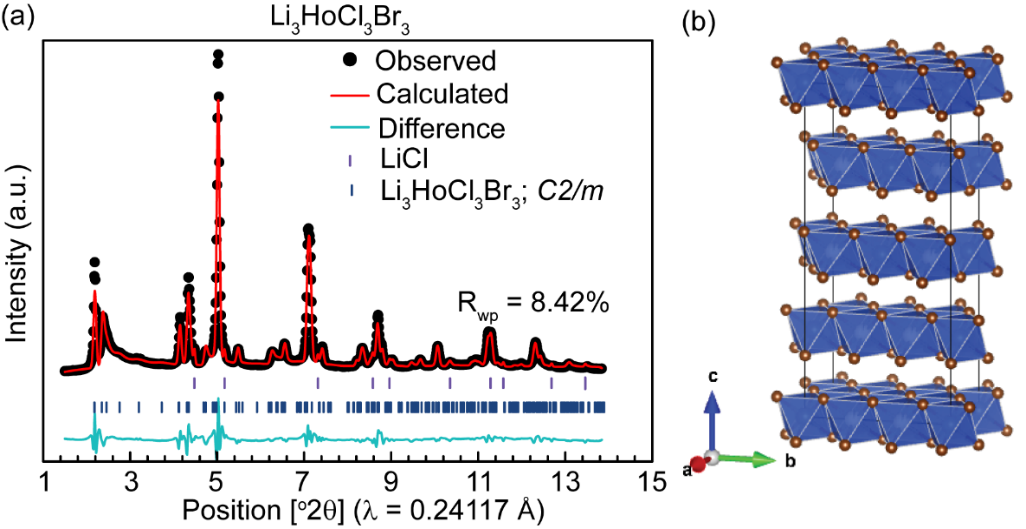


**Figure S1.** (a) Stacking faults Rietveld refinement of the high-resolution XRD data for Li_3_HoCl_3_Br_3_ using the Fault program in the Fullprof software (see Experimental Section for more details). Due to their low X-ray scattering power and negligible contribution to the XRD pattern, Li atoms were omitted from the refinement to reduce computational cost. (b) the refined structure of Li_3_HoCl_3_Br_3,_ showing the composition layers stacked in a randomly staggered fashion.


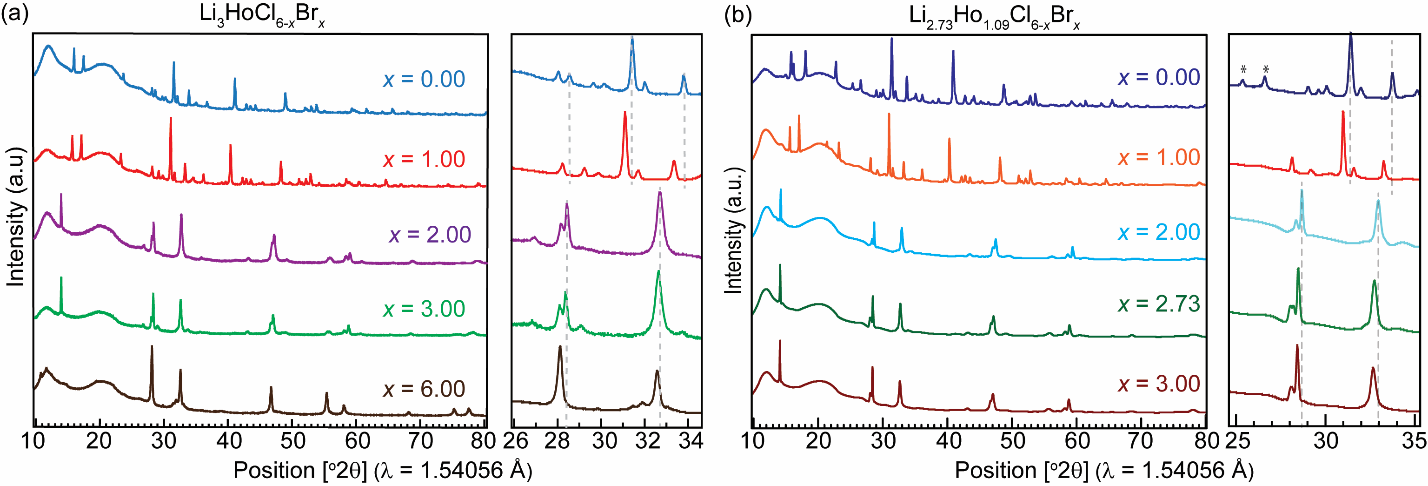


**Figure S2**. Powder X-ray diffraction patterns of (a) Li_3_HoCl_6-_*_x_*Br*_x_* (0 ≤ *x ≤* 3) and (b) Li_2.73_Ho_1.09_Cl_6-_*_x_*Br*_x_* (0 ≤ *x ≤* 2.73) series. The broad background peak around 20° is associated with the Kapton film used to seal the samples from air/moisture exposure. A magnified view of select 2θ range for each series is shown on their right side. A shift toward a lower 2θ value with increased bromination represents the lattice expansion.


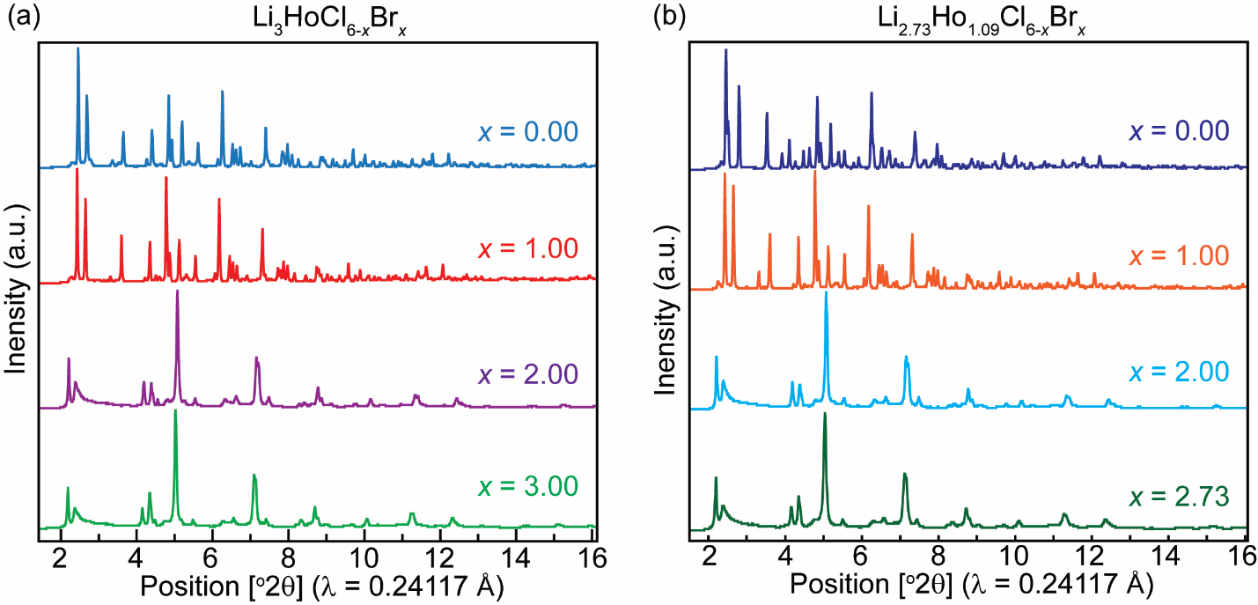


**Figure S3**. Comparison of high-resolution XRD patterns of (a) Li_3_HoCl_6-_*_x_*Br*_x_* (0 ≤ *x ≤* 3) and (b) Li_2.73_Ho_1.09_Cl_6-_*_x_*Br*_x_* (0 ≤ *x ≤* 2.73). For the Li_3_HoCl_6-_*_x_*Br*_x_* series, a phase transition from the P-3m1 space group for *x* = 0 and 1 to the C2/m space group for *x* = 2 and 3 is observed. Whereas, for Li_2.73_Ho_1.09_Cl_6-_*_x_*Br*_x_* series, a phase transition from Pnma for *x* = 0 to the P-3m1 space group for *x* = 1 and further to the C2/m space group for *x* = 2 and 3 is observed.


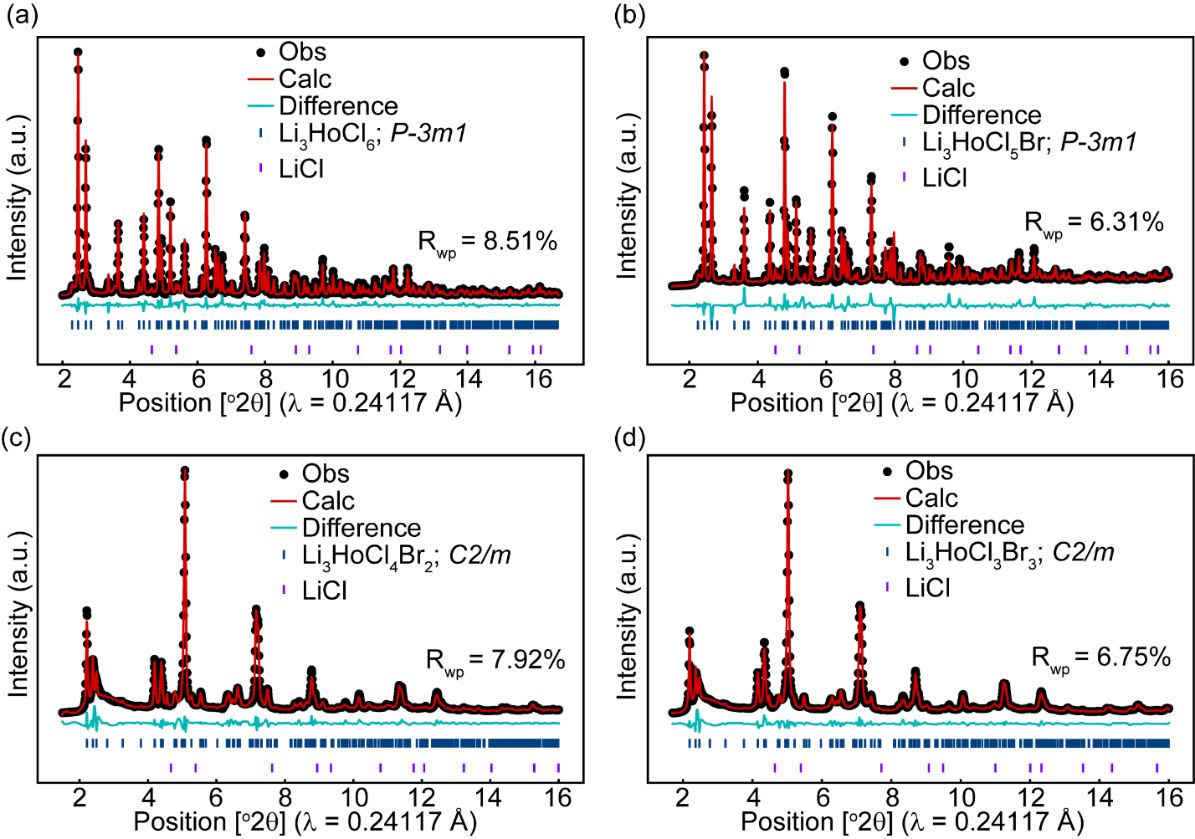


**Figure S4**. Rietveld refinement of the high-resolution X-ray diffraction patterns for the stoichiometric Li_3_HoCl_6-_*_x_*Br*_x_* (0 ≤ *x ≤* 3).


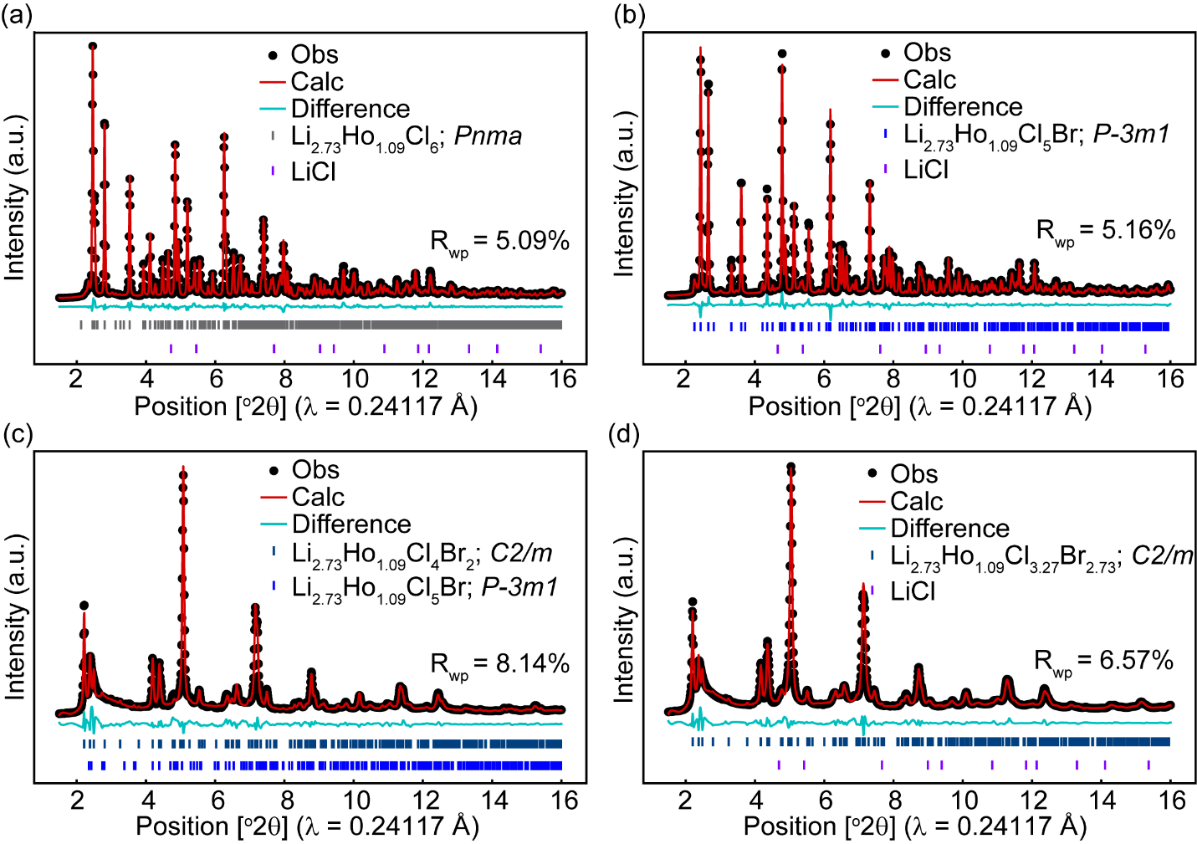


**Figure S5**. Rietveld refinement of the high-resolution X-ray diffraction patterns for the Li-deficient Li_2.73_Ho_1.09_Cl_6-_*_x_*Br*_x_* (0 ≤ *x ≤* 2.73).


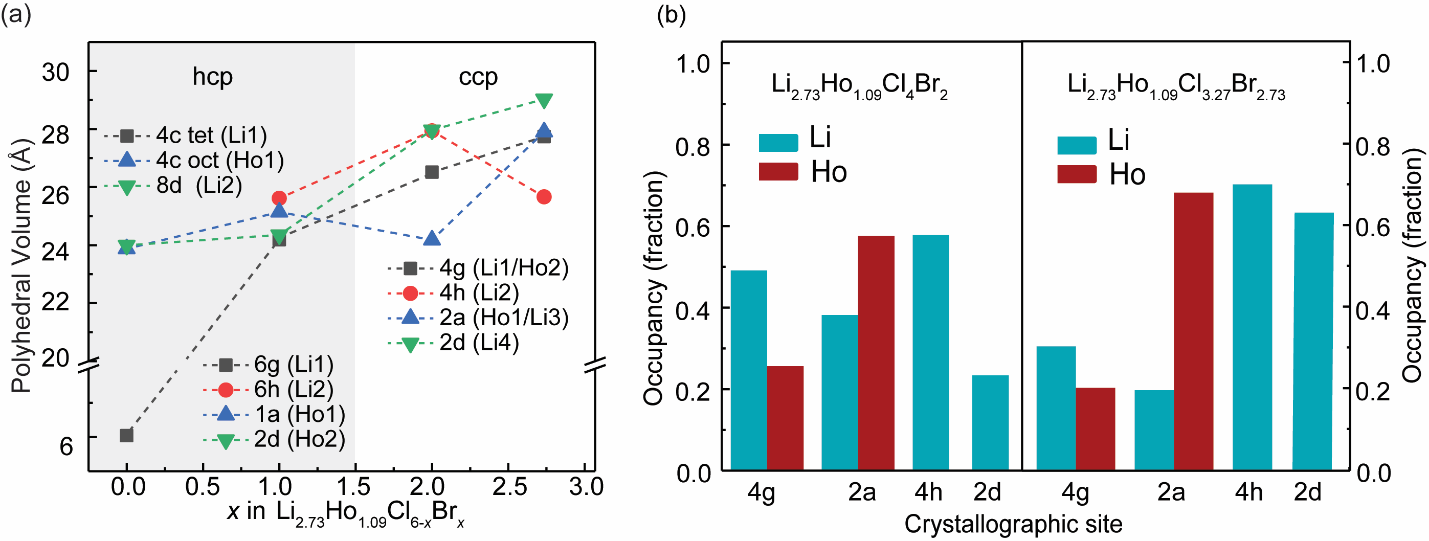


**Figure S6** (a) Polyhedral volume as a function of bromine substitution in Li_2.73_Ho_1.09_Cl_6-_*_x_*Br*_x_* (0 ≤ *x ≤* 2.73) shows a nonlinear increase in the octahedra sizes and (b) Cation site ordering for Li_2.73_Ho_1.09_Cl_4_Br_2_ and Li_2.73_Ho_1.09_Cl_3.27_Br_2.73_.


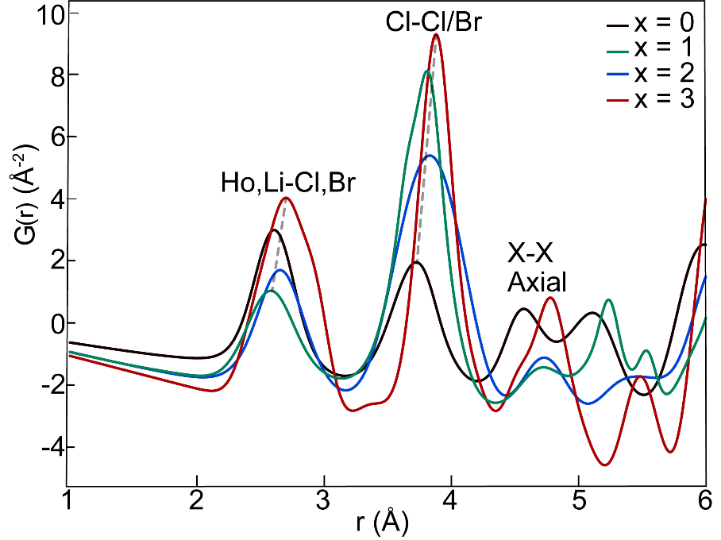


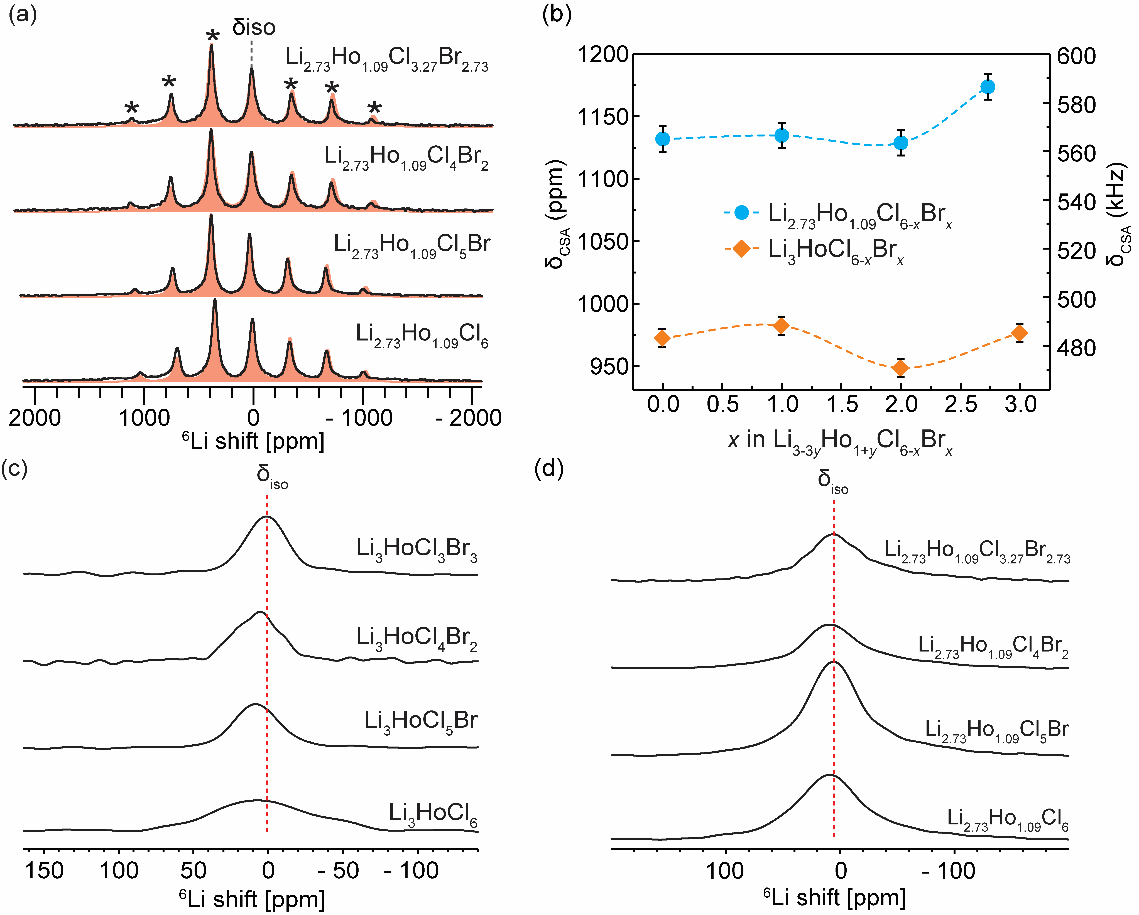
**Figure S7.** X-ray pair distribution function profile of Li_3_HoCl_6-x_Br*_x_* (0 ≤ *x ≤* 3). With the increase in the Br^-^ content, enhanced short-range disorder and increased distances of the Li-X (X = Cl/Br), Ho-X (X = Cl/Br), and Cl/Br-Cl/Br atom pairs are observed. The elongation of these bond pairs results in volume expansion of the octahedra, enhancing Li^+^ mobility.

**Figure S8**. Li local structural environments in Li_3-3_*_y_*Ho_1+_*_y_*Cl_6-_*_x_*Br*_x_* probed with high-resolution ^6^Li NMR. (a) ^6^Li NMR spectra of Li_2.73_Ho_1.09_Cl_6-x_Br_x_ (0 ≤ *x ≤* 2.73) (b) Chemical shift anisotropy as a function of x in Li_3_HoCl_6-_*_x_*Br*_x_* and Li_2.73_Ho_1.09_Cl_6-_*_x_*Br*_x_*.  Higher chemical shift anisotropy in the lithium-deficient series Li_2.73_Ho_1.09_Cl_6-x_Br_x_ indicates greater structural disorder, consistent with the X-ray diffraction refinement results. (c) Isotropic peak-focused ^6^Li NMR of Li_3_HoCl_6-_*_x_*Br*_x_* and (d) Isotropic peak-focused ^6^Li NMR of Li_2.73_Ho_1.09_Cl_6-x_Br_x_ series


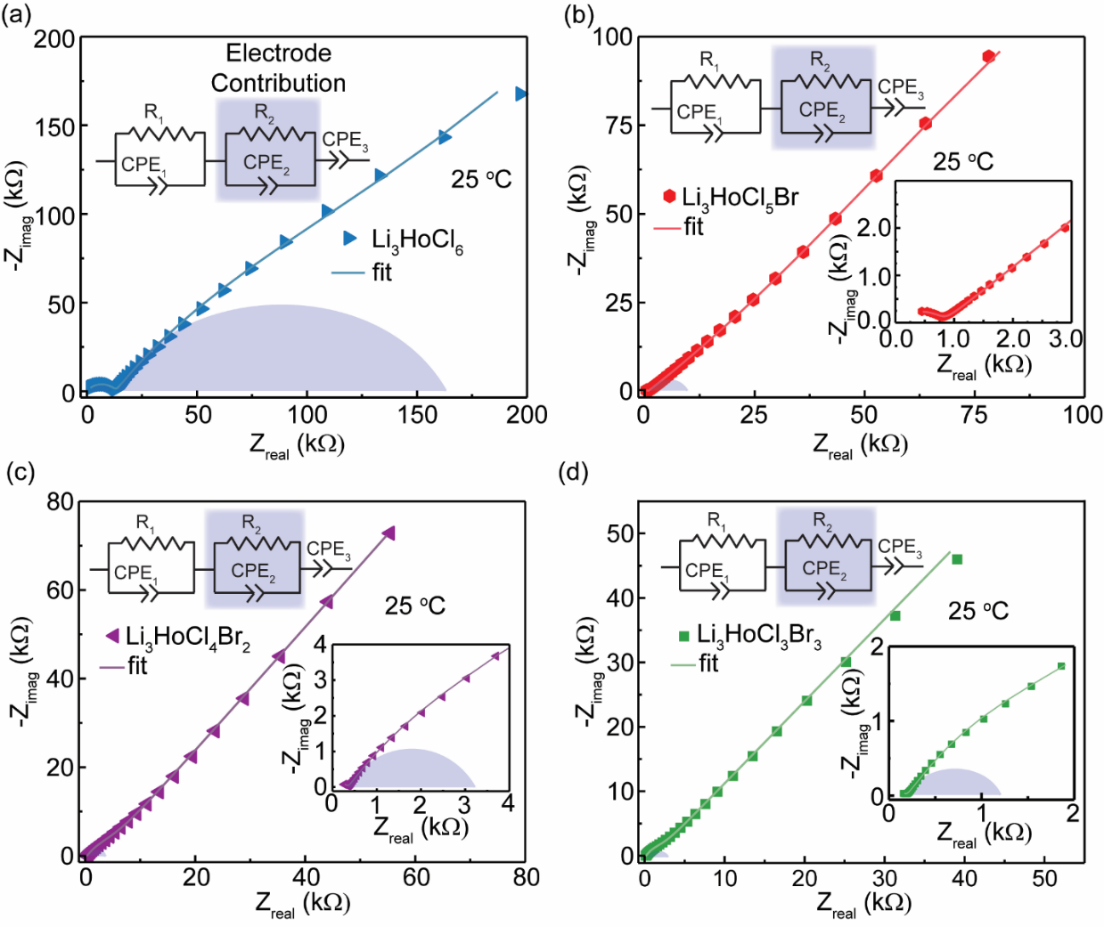


**Figure S9**. Nyquist plots of Li_3_HoCl_6-_*_x_*Br*_x_* at 25 ℃ and the corresponding equivalent circuit fitting.


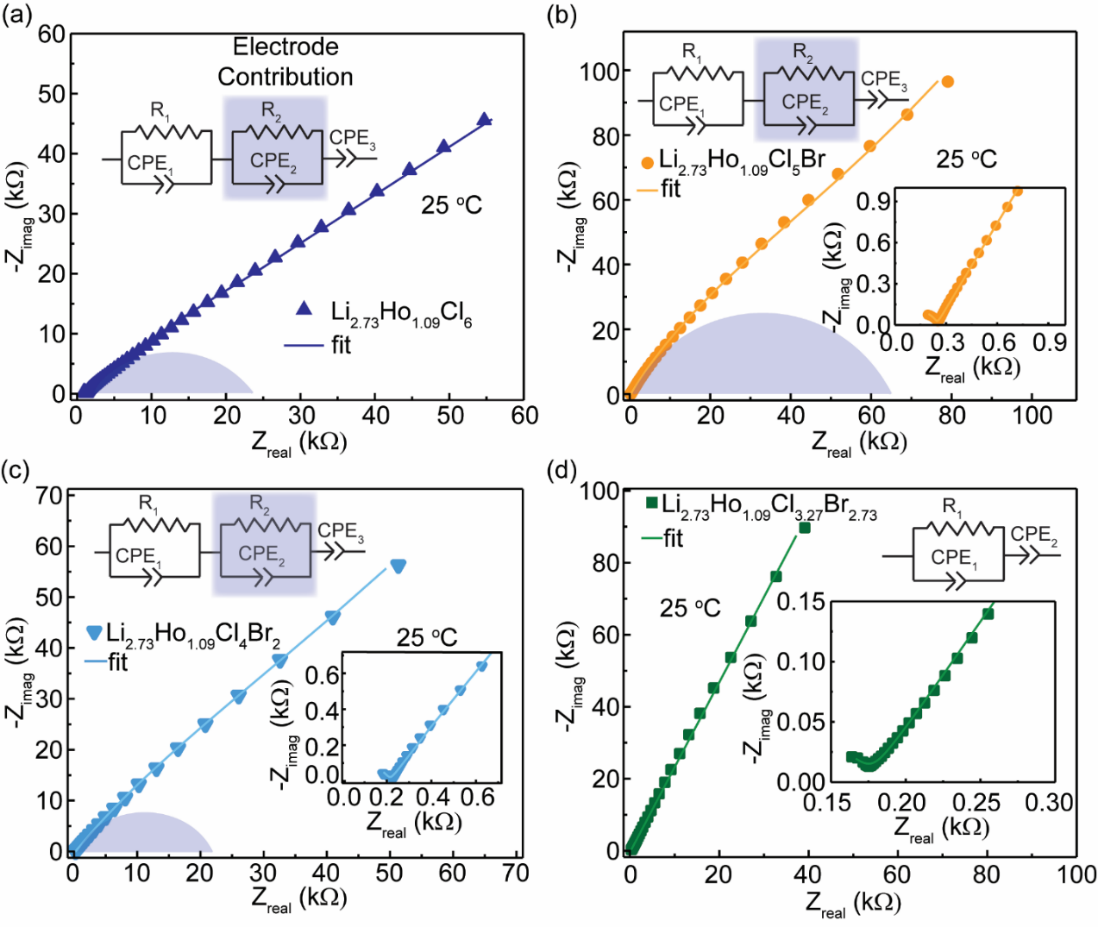


**
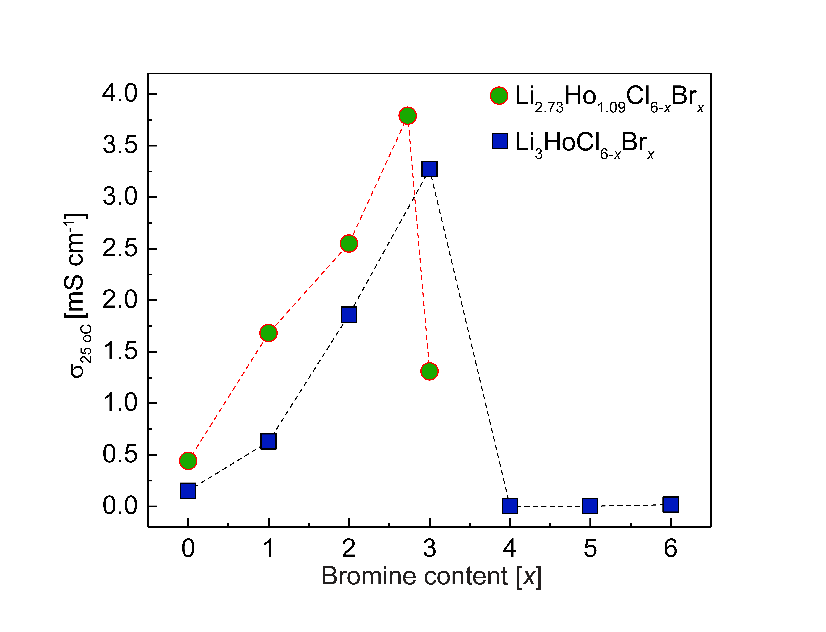
Figure S10**. Nyquist plots of Li_2.73_Ho_1.09_Cl_6-_*_x_*Br*_x_* at 25 ℃ and the corresponding equivalent circuit fitting.

**Figure S11.** Room-temperature ionic conductivity trend showing a decrease in conduction with higher bromine replacement.


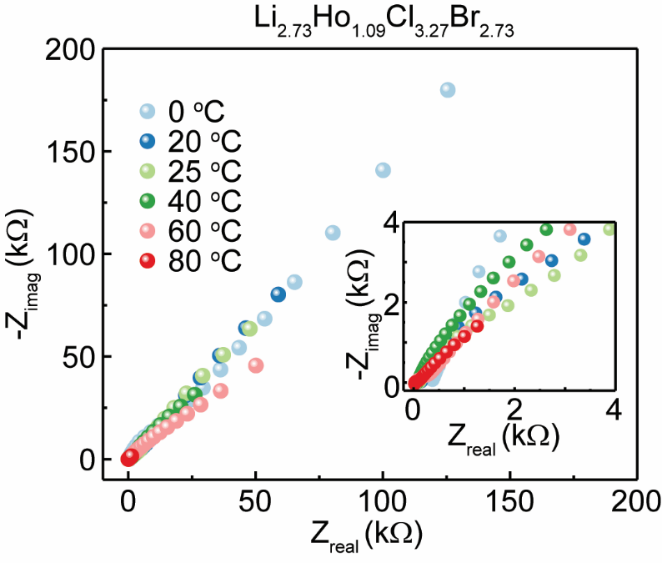


**Figure S12.** Select variable-temperature Nyquist plots measured in the temperature range from 0 ℃ to 80 ℃ for Li_2.73_Ho_1.09_Cl_3.27_Br_2.73_.

**Equation (S1).** The conductivity was calculated from the equivalent circuit model fits using the following equation,

σ_DC_ = $\frac{L}{R x A}$ (1)

where *L* and *A* are the thickness of the pellet and surface area of the blocking electrode respectively, and *R* is the value of resistance extracted from the equivalent circuit fitting.

**Equation (S2)**. The Arrhenius-type conductivity equation^1^ used is expressed as follows.

$\sigma_{DC}T=\sigma_{0}e^{-Ea/k_{B}T} (2)$

where σ_DC_ is the DC ionic conductivity, T is the temperature in K, σ_0_ is the Arrhenius perfector, E_a,_ is the activation energy, and k_B_ is the Boltzmann constant.


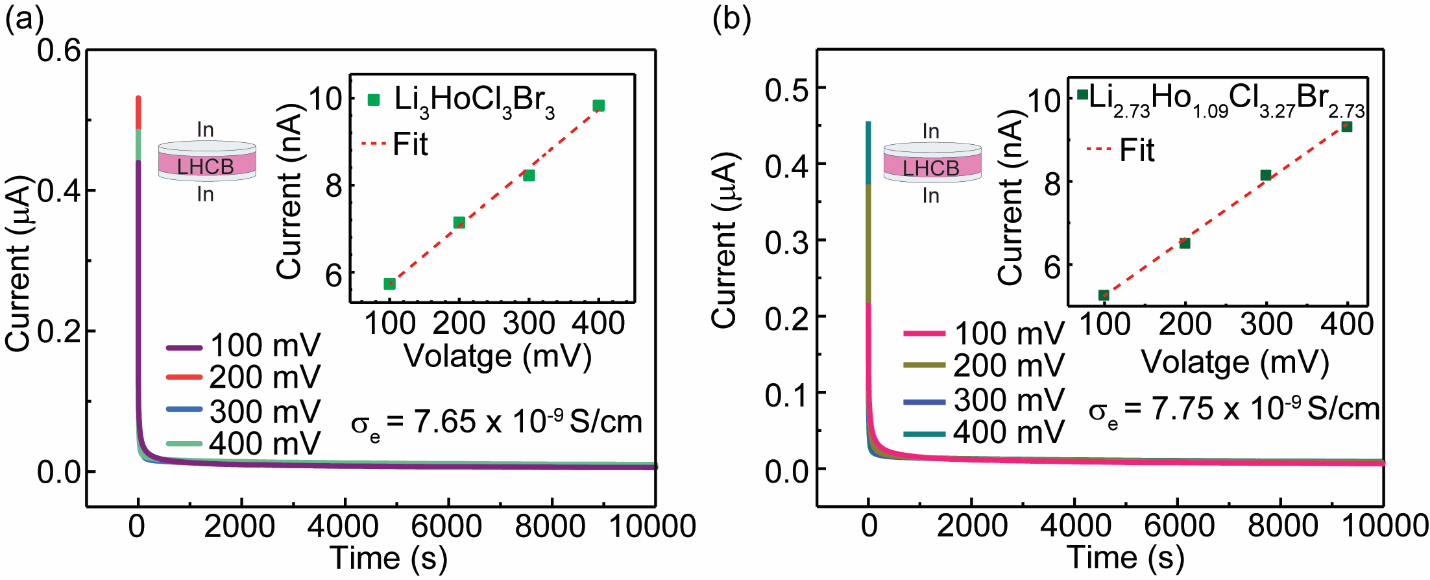


**Figure S13**. The electronic conductivities of Li_3_HoCl_3_Br_3_ (a) and Li_2.73_Ho_1.09_Cl_3.27_Br_2.73_ (b), were determined using the DC polarization technique. The inset displays the current *vs.* voltage graph from which the electronic resistance (Ohm's law) and electronic conductivities were derived.

**Supporting Tables**

**Table S1**. Rietveld-refinement results of high-resolution X-ray diffraction data at room temperature for the co-melted **Li_3_HoCl_6_**

Lattice parameter: a = 11.1964(6) b = 11. 1964(6), c = 6.0324(22), α = β = 90.000 γ = 120.000

Unit-cell volume = 654.91(4) Å^3^

Density = 3.031 g/cm^3^

R_wp_ = 8.51 %

Crystal system: Trigonal, Space group: *P-3m1*

Impurity phase: 1.60 % LiCl

| Name | Atom | Wycoff position | Atomic coordinates | | | Occupancy | U_iso_ |
| --- | --- | --- | --- | --- | --- | --- | --- |
|  |  |  | x | y | z |  |  |
| Li1 | Li | 6g | 0.364(1) | 0 | 0 | 0.6 | 0.177(8) |
| Li2 | Li | 6h | 0.322(7) | 0 | 0.5 | 0.9 | 0.103(3) |
| Ho1 | Ho | 1a | 0 | 0 | 0 | 1 | 0.010(1) |
| Ho2 | Ho | 2d | 0.333 | 0.667 | 0.5045(5) | 1 | 0.015(1) |
| Cl1 | Cl | 6i | 0.1128(3) | -0.1128(3) | 0.7715(8) | 1 | 0.012(2) |
| Cl2 | Cl | 6i | 0.2198(3) | -0.2198(3) | 0.2570(7) | 1 | 0.004(2) |
| Cl3 | Cl | 6i | 0.4405(4) | -0.4405(4) | 0.7670(8) | 1 | 0.027(2) |

**Table S2**. Rietveld-refinement results of the high-resolution X-ray diffraction data at room temperature for the co-melted **Li_3_HoCl_5_Br**

Lattice parameter: a = 11.3305(8) b = 11.3305(8), c = 6.1272(3), α = β = 90.000 γ = 120.000

Unit-cell volume = 681.23(5)Å^3^

Density = 3.240 g/cm^3^

R_wp_ = 6.31 %

Crystal system: Trigonal, Space group: *P-3m1*

Impurity phase: 0.2 % LiCl

| Name | Atom | Wycoff position | Atomic coordinates | | | Occupancy | U_iso_ |
| --- | --- | --- | --- | --- | --- | --- | --- |
|  |  |  | x | y | z |  |  |
| Li1 | Li | 6g | 0.380(7) | 0 | 0 | 0.8 | 0.046(9) |
| Li2 | Li | 6h | 0.299(1) | 0 | 0.5 | 0.7(1) | 0.229(7) |
| Ho1 | Ho | 1a | 0 | 0 | 0 | 1 | 0.021(1) |
| Ho2 | Ho | 2d | 0.333 | 0.667 | 0.497(1) | 1 | 0.029(1) |
| Br1 | Br | 6i | 0.113(1) | -0.113(1) | 0.770(3) | 0.167 | 0.005(4) |
| Cl1 | Cl | 6i | 0.113(1) | -0.113(1) | 0.770(3) | 0.833 | 0.005(4) |
| Br2 | Br | 6i | 0.226(1) | -0.226(1) | 0.272(2) | 0.167 | 0.002(3) |
| Cl2 | Cl | 6i | 0.226(1) | -0.226(1) | 0.272(2) | 0.833 | 0.002(3) |
| Br3 | Br | 6i | 0.450(1) | -0.450(1) | 0.782(2) | 0.167 | 0.063(5) |
| Cl3 | Cl | 6i | 0.450(1) | -0.450(1) | 0.782(2) | 0.833 | 0.063(5) |

**Table S3**. Rietveld-refinement results of the high-resolution X-ray diffraction data at room temperature for the co-melted **Li_3_HoCl_4_Br_2_**

Lattice parameter: a = 6.702(7), b = 11.573(1), c = 6.624(7), α = γ = 90.0000, β = 109.57(19),

Unit-cell volume; V = 484.13(6) Å^3^

Density; ρ = 3.343 g/cm^3^

R_wp_ = 7.92 %

Crystal system: Monoclinic, Space group: *C2/m*

Impurity phase: 0.1 % LiCl

| Name | Atom | Wycoff position | Atomic coordinates | | | Occupancy | U_iso_ |
| --- | --- | --- | --- | --- | --- | --- | --- |
|  |  |  | x | y | z |  |  |
| Li1 | Li | 4g | 0.5 | 0.837(1) | 0 | 0.617(8) | 0.067(1) |
| Li2 | Li | 4h | 0 | 0.164(3) | 0.5 | 0.322 | 0.218(8) |
| Li4 | Li | 2d | 0.5 | 0 | 0.5 | 0.976(2) | 0.218(8) |
| Li3 | Li | 2a | 0 | 0 | 0 | 0.147(5) | 0.012(1) |
| Ho1 | Ho | 2a | 0 | 0 | 0 | 0.707(5) | 0.012(1) |
| Ho2 | Ho | 4g | 0.5 | 0.837(1) | 0 | 0.147(3) | 0.067(1) |
| Br1 | Br | 4i | 0.234(2) | 0.8292(7) | 0.245(1) | 0.333 | 0.038(1) |
| Cl1 | Cl | 8j | 0.234(2) | 0.8292(7) | 0.245(1) | 0.667 | 0.038(1) |
| Br2 | Br | 4i | 0.739(3) | 0 | 0.227(2) | 0.333 | 0.010(2) |
| Cl2 | Cl | 8j | 0.739(3) | 0 | 0.227(2) | 0.667 | 0.010(2) |

**Table S4**. Rietveld-refinement results of the high-resolution X-ray diffraction data at room temperature for the co-melted **Li_3_HoCl_3_Br_3_**

Lattice parameter: a = 6.756(4), b = 11.683(15), c = 6.694(5), α = γ = 90.0000, β = 109.706(11),

Unit-cell volume; V = 497.46(7) Å^3^

Density; ρ = 3.532 g/cm^3^

R_wp_ = 6.75 %

Crystal system: Monoclinic, Space group: *C2/m*

Impurity phase: 0.2 % LiCl

| Name | Atom | Wycoff position | Atomic coordinates | | | Occupancy | U_iso_ |
| --- | --- | --- | --- | --- | --- | --- | --- |
|  |  |  | x | y | z |  |  |
| Li1 | Li | 4g | 0.5 | 0.856(5) | 0 | 0.901 | 0.007(4) |
| Li2 | Li | 4h | 0 | 0.164(2) | 0.5 | 0.478(9) | 0.059(5) |
| Li4 | Li | 2d | 0.5 | 0 | 0.5 | 0.044 | 0.059(5) |
| Li3 | Li | 2a | 0 | 0 | 0 | 0.198 | 0.024(2) |
| Ho1 | Ho | 2a | 0 | 0 | 0 | 0.802(5) | 0.024(2) |
| Ho2 | Ho | 4g | 0.5 | 0.8536(2) | 0 | 0.099(3) | 0.007(4) |
| Br1 | Br | 4i | 0.254(1) | 0.8332(6) | 0.2440(9) | 0.5 | 0.038(1) |
| Cl1 | Cl | 8j | 0.254(1) | 0.8332(6) | 0.2440(9) | 0.5 | 0.038(1) |
| Br2 | Br | 4i | 0.739(2) | 0 | 0.242(1) | 0.5 | 0.010(2) |
| Cl2 | Cl | 8j | 0.739(2) | 0 | 0.242(1) | 0.5 | 0.010(2) |

**Table S5**. Rietveld-refinement results of the high-resolution X-ray diffraction data at 300 K for the co-melted Li_2.73_Ho_1.09_Cl_6_.

Lattice parameter: a = 12.9733(3) b = 11.1750(3), c = 6.0426(1), α = β = γ = 90.0000

Unit-cell volume = 876.03(3) Å^3^

Density = 3.12 g/cm^3^

R_wp_ = 5.09%,

Crystal system: Orthorhombic, Space group: *Pnma*

Impurity phases: No impurity phase

| Name | Atom | Wycoff position | Atomic coordinates | | | Occupancy | U_iso_ |
| --- | --- | --- | --- | --- | --- | --- | --- |
|  |  |  | x | y | z |  |  |
| Li1 | Li | 8d | 0.108(1) | 0.061(2) | 0.027(3) | 0.865 | 0.152(9) |
| Li2 | Li | 4c | 0.198(6) | 0.25 | 0.61(1) | 1 | 0.08(1) |
| Ho1 | Ho | 8d | 0.108(1) | 0.061(2) | 0.027(3) | 0.045 | 0.152(9) |
| Ho2 | Ho | 4c | 0.3765(1) | 0.25 | 0.0127((5) | 1 | 0.0174(4) |
| Cl1 | Cl | 8d | 0.4578(9) | 0.079(1) | 0.2493(9) | 1 | 0.0242(7) |
| Cl2 | Cl | 8d | 0.2120(9) | 0.585(1) | 0.2709(9) | 1 | 0.0242(7) |
| Cl3 | Cl | 4c | 0.204(1) | 0.25 | 0.234(1) | 1 | 0.0242(7) |
| Cl4 | Cl | 4c | 0.048(1) | 0.25 | 0.728(1) | 1 | 0.0242(7) |

**Table S6**. Rietveld-refinement results of the high-resolution X-ray diffraction data at room temperature for the co-melted Li_2.73_Ho_1.09_Cl_5_Br.

Lattice parameter: a = b = 11.3280(4), c = 6.1199(2), α = β = 90.0000, γ = 120, Unit-cell volume = 680.11(3) Å^3^

Density = 3.35 g/cm^3^

R_wp_ = 5.16%,

Crystal System: Trigonal, Space group: *P-3m1*

Impurity phase: 0.6 wt% of LiCl

| Name | Atom | Wycoff position | Atomic coordinates | | | Occupancy | U_iso_ |
| --- | --- | --- | --- | --- | --- | --- | --- |
|  |  |  | x | y | z |  |  |
| Li1 | Li | 6g | 0.343(6) | 0 | 0 | 0.745 | 0.137(8) |
| Li2 | Li | 6h | 0.308(5) | 0 | 0.5 | 0.62(5) | 0.137(8) |
| Ho1 | Ho | 1a | 0 | 0 | 0 | 1 | 0.0235(8) |
| Ho2 | Ho | 2d | 0.333 | 0.667 | 0.518((1) | 1 | 0.0235(8) |
| Ho3 | Ho | 6g | 0.343(6) | 0 | 0 | 0.030(2) | 0.137(8) |
| Ho4 | Ho | 2d | 0.333 | 0.667 | 0.026(1) | 0.06 | 0.0235(8) |
| Cl1 | Cl | 6i | 0.112(1) | -0.112(1) | 0.7557(9) | 0.833 | 0.0235(5) |
| Cl2 | Cl | 6i | 0.224(1) | -0.224(1) | 0.281(1) | 0.833 | 0.0235(5) |
| Cl3 | Cl | 6i | 0.448(1) | -0.448(1) | 0.753(1) | 0.833 | 0.0235(5) |
| Br1 | Br | 6i | 0.112(1) | -0.112(1) | 0.7557(9) | 0.167 | 0.0235(5) |
| Br2 | Br | 6i | 0.224(1) | -0.224(1) | 0.281(1) | 0.167 | 0.0235(5) |
| Br3 | Br | 6i | 0.448(1) | -0.448(1) | 0.753(1) | 0.167 | 0.0235(5) |

**Table S7**. Rietveld-refinement results of the high-resolution X-ray diffraction data at room temperature for the co-melted Li_2.73_Ho_1.09_Cl_4_Br_2_.

Lattice parameter: a = 6.693(9), b = 11.586(2), c = 6.613(8), α = γ = 90.0000, β = 109.59(2),

Unit-cell volume; V = 483.14(9) Å^3^

Density; ρ = 3.428 g/cm^3^

R_wp_ = 8.14 %,

Crystal System: Monoclinic, Space group: C2/m

Impurity phase: 4 wt% of Li_2.73_Ho_1.09_Cl_5_Br

| Name | Atom | Wycoff position | Atomic coordinates | | | Occupancy | U_iso_ |
| --- | --- | --- | --- | --- | --- | --- | --- |
|  |  |  | x | y | z |  |  |
| Li1 | Li | 4g | 0.5 | 0.8552 (6) | 0 | 0.48(7) | 0.021(1) |
| Li2 | Li | 4h | 0 | 0.155(4) | 0.5 | 0.58 | 0.05(3) |
| Li3 | Li | 2d | 0.5 | 0 | 0.5 | 0.23(9) | 0.05(3) |
| Li4 | Li | 2a | 0 | 0 | 0 | 0.38(9) | 0.021(1) |
| Ho1 | Ho | 2a | 0 | 0 | 0 | 0.575 | 0.021(1) |
| Ho2 | Ho | 4g | 0.5 | 0.8552 (6) | 0 | 0.256(3) | 0.021(1) |
| Br1 | Br | 4i | 0.778(2) | 0 | 0.241(2) | 0.333 | 0.0213(9) |
| Br2 | Br | 8j | 0.243(1) | 0.8370(4) | 0.254(1) | 0.333 | 0.0213(9) |
| Cl1 | Br | 4i | 0.778(2) | 0 | 0.241(2) | 0.667 | 0.0213(9) |
| Cl2 | Br | 8j | 0.243(1) | 0.8370(4) | 0.254(1) | 0.667 | 0.0213(9) |

**Table S8**. Rietveld-refinement results of the high-resolution X-ray diffraction data at room temperature for the co-melted Li_2.73_Ho_1.09_Cl_3.27_Br_2.73_.

Lattice parameter: a = 6.737(3), b = 11.624(1), c = 6.669(4), α = γ = 90.0000, β = 109.797(8),

Unit-cell volume; V = 491.26(6) Å^3^

Density; ρ = 3. 591 g/cm^3^

R_wp_ = 6.57 %,

Crystal system: Monoclinic, Space group: C2/m

Impurity phases: 0 wt% of LiBr; 0.7 wt% of LiCl; 0wt% of HoCl_3_

| Name | Atom | Wycoff position | Atomic coordinates | | | Occupancy | | U_iso_ |
| --- | --- | --- | --- | --- | --- | --- | --- | --- |
|  |  |  | x | y | z |  |  |  |
| Li1 | Li | 4g | 0.5 | 0.8539(9) | 0 | | 0.30(4) | 0.030(1) |
| Li2 | Li | 4h | 0 | 0.15256(4) | 0.5 | | 0.70 | 0.032(5) |
| Li3 | Li | 2d | 0.5 | 0 | 0.5 | | 0.63(8) | 0.032(5) |
| Li4 | Li | 2a | 0 | 0 | 0 | | 0.10(1) | 0.030(1) |
| Ho1 | Ho | 2a | 0 | 0 | 0 | | 0.688 | 0.030(1) |
| Ho2 | Ho | 4g | 0.5 | 0.8539(9) | 0 | | 0.201(3) | 0.030(1) |
| Br1 | Br | 4i | 0.747(1) | 0 | 0.236(1) | | 0.455 | 0.0212(8) |
| Br2 | Br | 8j | 0.2562(7) | 0.8260(3) | 0.2458(8) | | 0.455 | 0.0212(8) |
| Cl1 | Br | 4i | 0.747(1) | 0 | 0.236(1) | | 0.545 | 0.0212(8) |
| Cl2 | Br | 8j | 0.2562(7) | 0.8260(3) | 0.2458(8) | | 0.545 | 0.0212(8) |

**Table S9**. Summary of Li NMR analysis on Li_3_HoCl_6-_*_x_*Br*_x_* and Li_2.73_Ho_1.09_Cl_6-_*_x_*Br*_x_*.

| Series | Bromine content (*x*) | ^7^Li, *T*_1_  [ms] | ^6^Li, *δ*_iso_  [ppm] | ^6^Li, *δ*_CSA_  [ppm] | ^6^Li, *δ*_CSA_  [kHz] |
| --- | --- | --- | --- | --- | --- |
| Li_3_HoCl_6-_*_x_*Br*_x_* | 0 | 1.67(1) | 8.00 | 972 ± 7.5 | 486 ± 3.3 |
|  | 1 | 0.97(1) | 7.98 | 982 ± 7.5 | 491 ± 3.3 |
|  | 2 | 0.843(3) | 7.15 | 948 ± 7.5 | 474 ± 3.3 |
|  | 3 | 0.831(3) | 2.29 | 976 ± 7.5 | 488 ± 3.3 |
| Li_2.73_Ho_1.09_Cl_6-_*_x_*Br*_x_* | 0 | 1.461(5) | 7.04 | 1130 ± 11 | 565.0 ± 5.4 |
|  | 1 | - | 5.51 | 1133 ± 11 | 566.5 ± 5.4 |
|  | 2 | - | 4.06 | 1127 ± 11 | 563.5 ± 5.4 |
|  | 2.73 | 0.830(4) | 2.80 | 1173 ± 11 | 586.5 ± 5.4 |

**Table S10**. Summary of EIS analysis on Li_3_HoCl_6-_*_x_*Br*_x_* and Li_2.73_Ho_1.09_Cl_6-_*_x_*Br*_x_* including ionic conductivity, activation energy, and Arrhenius prefactor.

| Series | Bromine content (*x*) | *σ*_25 ℃_  [mS cm^-1^] | *E*_a_  [eV] | Log(*σ*_0_)  [S cm^-1^ K] |
| --- | --- | --- | --- | --- |
| Li_3_HoCl_6-_*_x_*Br*_x_* | 0 | 0.16 ± 0.04 | 0.507 ± 0.012 | 6.54 ± 0.06 |
|  | 1 | 0.63 ± 0.05 | 0.409 ± 0.002 | 6.12 ± 0.06 |
|  | 2 | 1.86 ± 0.36 | 0.358 ± 0.006 | 5.77 ± 0.05 |
|  | 3 | 3.27 ± 0.27 | 0.336 ± 0.012 | 5.61 ± 0.13 |
| Li_2.73_Ho_1.09_Cl_6-_*_x_*Br*_x_* | 0 | 0.44 ± 0.08 | 0.395 ± 0.010 | 5.93 ± 0.10 |
|  | 1 | 1.68 ± 0.18 | 0.435 ± 0.004 | 6.95 ± 0.06 |
|  | 2 | 2.55 ± 0.26 | 0.382 ± 0.011 | 6.32 ± 0.09 |
|  | 2.73 | 3.79 ± 0.54 | 0.322 ± 0.005 | 5.57 ± 0.09 |

**Reference**

[1.] R. B. Nuernberg, Ionics 2020, 26, 2405.
